# Distinct but cooperating brain networks supporting semantic cognition

**DOI:** 10.1093/cercor/bhac190

**Published:** 2022-05-21

**Authors:** JeYoung Jung, Matthew A Lambon Ralph

**Affiliations:** School of Psychology, University of Nottingham, Nottingham NG7 2RD, United Kingdom; MRC Cognition and Brain Science Unit (CBU), University of Cambridge, Cambridge, CB2 7EF United Kingdom

**Keywords:** semantic cognition, functional brain networks, independent component analysis (ICA), default mode network (DMN), frontoparietal network (FPN)

## Abstract

Semantic cognition is a complex multifaceted brain function involving multiple processes including sensory, semantic, and domain-general cognitive systems. However, it remains unclear how these systems cooperate with each other to achieve effective semantic cognition. Here, we used independent component analysis (ICA) to investigate the functional brain networks that support semantic cognition. We used a semantic judgment task and a pattern-matching control task, each with 2 levels of difficulty, to disentangle task-specific networks from domain-general networks. ICA revealed 2 task-specific networks (the left-lateralized semantic network [SN] and a bilateral, extended semantic network [ESN]) and domain-general networks including the frontoparietal network (FPN) and default mode network (DMN). SN was coupled with the ESN and FPN but decoupled from the DMN, whereas the ESN was synchronized with the FPN alone and did not show a decoupling with the DMN. The degree of decoupling between the SN and DMN was associated with semantic task performance, with the strongest decoupling for the poorest performing participants. Our findings suggest that human higher cognition is achieved by the multiple brain networks, serving distinct and shared cognitive functions depending on task demands, and that the neural dynamics between these networks may be crucial for efficient semantic cognition.

## Introduction

Human higher cognition is supported by a task-dependent dynamic configuration of functional brain networks. To achieve successful cognition, functional brain networks constantly reconfigure their architecture in response to the changing cognitive demands, and this leads to functional segregation and integration of brain networks (Bressler and Kelso 2001). Importantly, our cognitive performance links to the dynamic reorganization of functional brain networks (Cohen and D'Esposito 2016; Shine et al. 2016). Thus, it is an important challenge for cognitive neuroscience to understand the dynamics of functional brain networks in relation to cognitive demands. Here, we explored this issue targeting semantic cognition as our cognitive domain of interest given that it is a core higher cognitive function involving multiple complex processes.

Semantic cognition refers to our ability to use, manipulate, and generalize knowledge in order to interact with the world, by producing time- and context-appropriate behaviors (Binder et al. 2009; Lambon Ralph et al. 2017). It requires a complex brain function involving multiple processes spanning sensory systems (Binder et al. 2016; Martin 2016), semantic representation (Binney et al. 2010; Lambon Ralph et al. 2010b; Chen et al. 2016), semantic control processes (Whitney et al. 2011; Noonan et al. 2013), and domain-general cognitive systems (Raichle et al. 2001; Duncan 2010; Vossel et al. 2014). In addition to task-active systems, the default mode network (DMN), a task negative system, has also been associated with semantic cognition (Binder et al. 1999). Although these distinctive systems are involved in semantic processing, it remains unclear how these systems cooperate with each other to achieve effective semantic cognition.

Lambon Ralph et al. (2017) proposed the “controlled semantic cognition” (CSC) framework that incorporates 2 key systems for semantic cognition: semantic representation and control. In CSC, semantic representation is supported by a distributed system consisting of a transmodal hub interacting with multiple modality-specific spokes. Empirical and computational evidence has demonstrated that the bilateral anterior temporal lobes (ATLs) are a site for the transmodal hub (Coccia et al. 2004; Binney et al. 2010; Peelen and Caramazza 2012). Damage to this region incurs a severe degradation of semantic knowledge observed across all modalities and types of concept (Bozeat et al. 2000; Patterson et al. 2007). The semantic control system guides the representational system to select and shape a particular concept or generate a proper behavior in a given task or context. For example, if you use a keyboard, you need to know its function as an input device to enter characters and generate a proper action, *typing*. However, if you were asked to move the keyboard, you must ignore its main function and produce a different set of behaviors such as *grasping and carrying*. The control system is implemented in a distributed network including the left inferior frontal gyrus (IFG), posterior middle temporal gyrus (pMTG), and intraparietal sulcus (IPS) (Thompson-Schill et al. 1997; Badre and Wagner 2002; Noonan et al. 2013). Damage to this system, as observed in semantic aphasia, produces difficulty in controlling conceptual retrieval to suit a specific task or context (Jefferies and Lambon Ralph 2006; Corbett et al. 2009).

The semantic control system overlaps with the frontoparietal network (FPN). The FPN is involved in cognitive control across domains and responds to a wide range of demanding cognitive tasks (Duncan 2010; Fedorenko et al. 2012). The FPN includes the bilateral dorsolateral prefrontal cortex (DLPFC), inferior frontal sulcus (IFS), anterior cingulate cortex (ACC), pre/supplementary motor area (preSMA/SMA), and IPS. It is possible that semantic control calls upon both domain-specific (semantic) control mechanisms as well as domain-general executive control processing. Researchers have attempted to delineate these 2 systems and have proposed a superior–inferior functional specialization of the FPN. Functional MRI studies have reported that the ventral PFC (vPFC) and pMTG show increased activation during the retrieval of weak semantic association, whereas the activation of DLPFC and IPS varies in line with selection demands regardless of task (Badre et al. 2005; Nagel et al. 2008). Studies of functional connectivity have also shown that the vPFC and pMTG are connected to the ATL, suggesting their role of the regulation of semantic representation, whereas superior parts of the network are not (Jackson et al. 2016; Jung et al. 2018; Jung et al. 2022). Although studies have demonstrated a role of FPN in semantic cognition, it still remains unclear how semantic processing reshapes the FPN.

Previous fMRI studies have shown that the semantic network (SN) sometimes overlaps with the DMN (Binder et al. 1999; Binder et al. 2009; Wirth et al. 2011; Humphreys et al. 2015; Jackson et al. 2016). The DMN is localized primarily to midline anterior and posterior cortical regions, angular gyrus (AG), and medial and lateral temporal cortices (Raichle et al. 2001). It normally shows activation at rest and deactivation during goal-directed tasks, and has been associated with autobiographical memory retrieval, self-related thinking, and consciousness (Buckner et al. 2008). The involvement of the DMN in semantic processing is currently unclear and debated. As noted previously, fMRI participants during “rest” probably engage in spontaneous language and semantic processing, and thus, this commonality could lead to the overlapping brain regions for the DMN and semantic system (Binder et al. 1999; Humphreys et al. 2015). In addition, some studies have found that the DMN showed less deactivation during semantic processing compared to nonsemantic processing, specifically within the left-hemispheric DMN regions including the ATL, AG, medial PFC (mPFC), pMTG, and retrosplenial cortex (Wirth et al. 2011). More recent fMRI studies, designed to reliably probe ATL activations, have demonstrated different functions for the overlapping regions between the DMN and SN (Humphreys et al. 2015). The ATL was activated for all semantic tasks but deactivated for nonsemantic tasks, whereas the AG was deactivated for all tasks, showing greater deactivation for more demanding tasks (Humphreys and Lambon Ralph 2015, 2017; Humphreys et al. 2020; Humphreys et al. 2022). These studies suggest a functional segregation of the DMN during semantic processing, but it is still poorly understood how these 2 systems interact with each other.

Independent component analysis (ICA) is a processing technique to individuate signals from a mixture of sources (McKeown and Sejnowski 1998; Calhoun et al. 2001b). ICA assumes that the fMRI signal from each voxel represents a linear mixture of sources. It separates this mixture into independent source signals and groups all voxels into independent components (ICs), each of which represents temporally coherent functional brain networks. Several studies have demonstrated that ICA can reveal more brain regions involved in tasks than traditional general linear model (GLM) analysis and also can identify different task-related modulations in overlapping regions of 2 or more functional networks (Calhoun et al. 2001a; Kim et al. 2009; Domagalik et al. 2012; Geranmayeh et al. 2014). Geranmayeh et al. (2014) identified overlapping networks during spoken language production and then specified their involvement in speech processing, using ICA. Therefore, this approach can be more sensitive to detect task-related brain activity and delineate specific involvement of overlapping regions according to tasks.

Here, we performed an fMRI study to test for the presence of functionally independent but spatially overlapping brain networks for semantic cognition. We used a semantic judgment task and a pattern-matching control task, each with 2 levels of difficulty, in order to disentangle domain-specific (semantic) processing from domain-general processing (nonsemantic) and to delineate task-specific involvement of overlapping brain networks during semantic processing. We hypothesized that (i) ICA would identify several networks related to semantic processing including the semantic system, FPN, and DMN and (ii) task difficulty modulation would segregate or integrate these networks. In order to investigate the functional interaction between these networks during semantic processing, we performed functional network connectivity (FNC) analysis. Finally, we explored the relationship between the internetwork connectivity and semantic task performance.

## Methods

### Participants

Twenty-three healthy young participants were recruited for this study (12 females, mean age = 21 ± 3 years ranging from 19 to 30 years). They were English native speakers with right-handedness (Oldfield 1971) and had normal or corrected-normal vision. All participants gave their written informed consent prior to the study. The experiment was approved by local ethics committee in accordance with the Declaration of Helsinki.

### Experimental design and procedure

Participants performed a semantic judgment task and a pattern matching task (a control task) with 2 levels of difficulty (easy vs. hard) during fMRI. The semantic judgment task was adapted from a previous fMRI study (Jackson et al. 2015). Participants were presented with triads of concrete nouns and asked to judge which of the 2 choices was more related to the probe word. In each trial, 3 words were presented on the screen, a probe on the top and 2 choices (a target and a foil) at the bottom. The level of difficulty was modulated by 2 different foils. One foil was from an unrelated category to the probe word (easy condition) (e.g. CARROT [probe]—GRAPE [target] paired with the foil, TELESCOPE). The other foil was from the same or a related category to the target and probe (hard condition) (e.g. CARROT [probe]—GRAPE [target] paired with the foil, BLUBELL). Targets, probes, and foils were matched for frequency, imageability, letter length, and syllable length (*P*s > 0.5). A pattern matching task was also adapted from a previous study (Jung et al. 2021). The items for the control task were generated by visually scrambling items from the semantic task. Each pattern was created by scrambling each item into 120 pieces and rearranging them in a random order. Participants were asked to select which of 2 patterns was identical to a probe pattern. In the hard condition, the target patterns were presented 180° rotated.

In the scanner, stimuli were presented in a block design and there were 4 task blocks from each condition (semantic easy [SE], semantic hard [SH], control easy [CE], and control hard [CH]). A task block had 4 trials from the same condition. Between the task blocks, there were fixation blocks for 4000 ms. Each trial started with a 500 ms fixation and was presented for 3000 ms. Participants responded by pressing one of 2 buttons representing the left and right options. The 4 task blocks were sampled 7 times, giving a total of 28 trials for a condition. The semantic and control tasks were paired for the same difficulty level and presented in a counterbalanced order (e.g. A-B-A-B). The total time of scanning was about 8 min. E-prime software (Psychology Software Tools Inc., Pittsburgh, USA) was used to display stimuli and to record responses.

### Functional MRI data acquisition and GLM analysis

Imaging was performed on a 3 T Philips Achieva scanner using a 32-channel head coil with a SENSE factor 2.5. To maximize signal-to-noise in the ATL, we utilized a dual-echo fMRI protocol developed by Halai et al. (2014). The fMRI sequence included 42 slices, 96 × 96 matrix, 240 × 240 × 126 mm FOV, in-plane resolution 2.5 × 2.5, slice thickness 3 mm, TR = 2.8 s, TE = 12 ms and 35 ms. 180 dynamic scans were acquired. The structural image was acquired using a 3D MPRAGE pulse sequence with 200 slices, in planed resolution 0.94 × 0.94, slice thickness 0.9 mm, TR = 8.4 ms, and TE = 3.9 ms.

Analysis was carried out using SPM8 (Wellcome Department of Imaging Neuroscience, London; www.fil.ion.ucl.ac.uk/spm). The dual gradient echo images were extracted and averaged using in-house MATLAB code developed by Halai et al. (2014). Functional images were realigned correcting for motion artifacts and different signal acquisition times by shifting the signal measured in each slice relative to the acquisition of the middle slice prior to combining the short and long echo images. The mean functional image of echo-planar imaging (EPI) was co-registered to the individual T1-weighted image and segmented using the DARTEL (diffeomorphic anatomical registration through an exponentiated lie algebra) toolbox (Ashburner 2007). Then, normalization was performed using DARTEL to warp and reslice images into MNI space and smoothing was applied with an 8-mm full-width half-maximum Gaussian filter.

At the individual subject level, contrasts of interest were modeled using a box-car function convolved with the canonical hemodynamic response function. Four separate regressors were modeled according to task and difficulty (SE, SH, CE, and CH). At the group level, a 2-factorial analysis of variance (ANOVA) with task (semantic vs. control) and difficulty (easy vs. hard) was conducted for the main effect of task and interaction between task and difficulty and T-contrasts were established for the contrast of semantic > control and control > semantic. Whole-brain maps were thresholded at *P* < 0.001 at the voxel level, with an FWE-corrected cluster threshold of *P* < 0.05, ks > 100.

### Regions of interest (ROI) analysis

A prior ROI analysis was performed to evaluate the level of activation in regions associated with semantic processing and difficulty manipulation. Peak coordinates were taken from previous fMRI studies (Binney et al. 2010; Noonan et al. 2013; Humphreys et al. 2015; Jung et al. 2021) and included ATL [MNI: −36 −15 −30; 36 −15 30, IFG (pars. Orbitalis) [MNI: −45 27 −15; 45 27–15], pMTG [MNI: −66 −42 3; 66 −42 3], IFG (pars. Triangularis) [MNI: −45 19 18], AG [MNI −51 −72 24], ventromedial PFC (vmPFC) [MNI 0 51 −12], dorsomedial PFC (dmPFC) [MNI −3 42 48], precuneus [−3 −48 30], IPS [MNI −42 −42 42], and middle occipital gyrus (MOG) [MNI −39 −87 15]. Each ROI was created as a sphere with 8-mm radius. Paired *t*-tests were performed on contrast value (semantic > control) for key semantic ROIs (ATL, IFG, and pMTG) and signal changes for ROIs associated with difficulty manipulation across task conditions (*P*_FDR-corrected_ < 0.05).

### Independent component analysis

ICA is a data-driven multivariate approach to decompose a mixed signal into ICs (Calhoun et al. 2001b). ICA utilizes fluctuation in the fMRI data to separate the signal into maximally independent spatial maps (components), each explaining unique variance of the 4D fMRI data. Each component has a time course related to a coherent neural signaling associated with a specific task, artefact, or both.

ICA was performed using a group ICA algorithm (GIFT, http://icatb.sourceforge.net/, version 3.0a). Using Maximum Description Length and Akaike’s criteria, the number of ICs was estimated. A first stage subject-specific principal components analysis (PCA) was performed. A second stage group data reduction, using the expectation–maximization algorithm included in GIFT. Then, Informax ICA algorithm (Bell and Sejnowski 1995) was conducted, repeating it 20 times in ICASSO implemented in GIFT to generate a stable set of 30 final components. Finally, the ICs were then estimated using the GICA back-reconstruction method based on PCA compression and projection (Calhoun et al. 2001b).

Of the resultant 30 ICs, 15 components were related to residual artefact including the signal distributed around the edge of the brain and within cerebrospinal fluid spaces, variation in head size, or vascular blood flow (Supplementary Fig. S1). These were excluded for further analysis. The remaining 15 ICs had signal distributed within the brain and were defined as “networks.” We labeled them with regional or functional descriptors (e.g. DMN, FPN).

For each of the 15 components, we tested whether that component was significantly involved in any task condition, using the “temporal sort” of GIFT. Temporal sorting was conducted by applying a GLM to the component’s time course. The fMRI run-specific time courses for each subject were regressed against the design matrix for the tasks and tested for significance to identify components where activity was greater during semantic and control processing (easy and hard) than rest. The resulting beta (β) weights represent the degree to which component network recruitment was modulated by the task conditions. For a given component, positive and negative β weights indicate task-related network recruitment that is increased or decreased with respect to baseline, respectively. To evaluate the task-related network recruitment, we performed a one-sample *t*-test on the β weights (*P*_FDR-corrected_ < 0.005). A 2-factorial ANOVA with task (semantic vs. control) and difficulty (easy vs. hard) was conducted for the main effect of task and interaction between task and difficulty. For the comparison between easy and hard condition for each task, post hoc paired *t*-tests were performed for each component (*P* < 0.05).

We evaluated the spatial similarity between the components and GLM brain activation maps, using the “spatial sort” of GIFT. Spatial sorting was conducted on the components showed the significant task-relatedness in temporal sorting, by applying the thresholded GLM group results from the contrasts of interest to the component’s spatial maps. The resulting beta (β) weights represent the degree to which component network was overlapping with the GLM brain activation maps. To evaluate the spatial similarity, we performed a one-sample *t*-test on the β weights (*P*_FDR-corrected_ < 0.005). For the comparison between easy and hard condition for each task, paired *t*-tests were performed for each component (*P*_FDR-corrected_ < 0.05).

To assess the connectivity between networks, FNC was performed for the networks showed the significant temporal and spatial task modulations. FNC was estimated as the Pearson’s correlation coefficients between pairs of time-courses of networks (Jafri et al. 2008) and tested using one-sample *t*-test (*P*_FDR-corrected_ < 0.05). Then, to explore the relationship between the FNC and semantic performance, correlation analysis was performed (*P*_FDR-corrected_ < 0.05).

## Results

### Behavioral results

A repeated-measure ANOVA with task (semantic vs. control) and difficulty (easy vs. hard) was conducted for accuracy and reaction time (RT). In accuracy, there was a significant main effect of difficulty (*F*_1, 22_ = 18.34, *P* < 0.001). The other main effect and interaction did not reach the significance. In RT, the results showed a significant main effect of task (F_1, 22_ = 11.54, *P* < 0.005), difficulty (F_1, 22_ = 64.22, *P* < 0.001), and an interaction (F_1, 22_ = 24.67, *P* < 0.001). Post hoc paired *t*-tests revealed that the difficulty manipulation was successful in the accuracy and RT. The accuracy was significantly reduced (semantic: *t* = 6.23, *P* < 0.001; control: *t* = 2.35, *P* < 0.05) and the RT was significantly increased for the hard condition compared to the easy condition (semantic: *t* = −4.80, *P* < 0.001; control: *t* = −8.52, *P* < 0.001). There was no difference in the accuracy between semantic and control tasks (*P*s > 0.35). The RT showed no difference in the easy condition (*P* = 0.69) but a significant difference in the hard condition between the semantic and control tasks (*t* = −5.56, *P* < 0.001). The results are summarized in Table 1.

**Table 1:** Behavioral results. Mean (standard error).

	Semantic		Control	
	ACC	RT	ACC	RT
Easy	93.2 (1.6)	1411.2 (51.3)	90.8 (3.1)	1430.7 (48.9)
Hard	83.9 (1.8)	1603.4 (45.2)	81.3 (2.3)	1859.1 (40.9)

### Imaging results

**Fig. 1:**
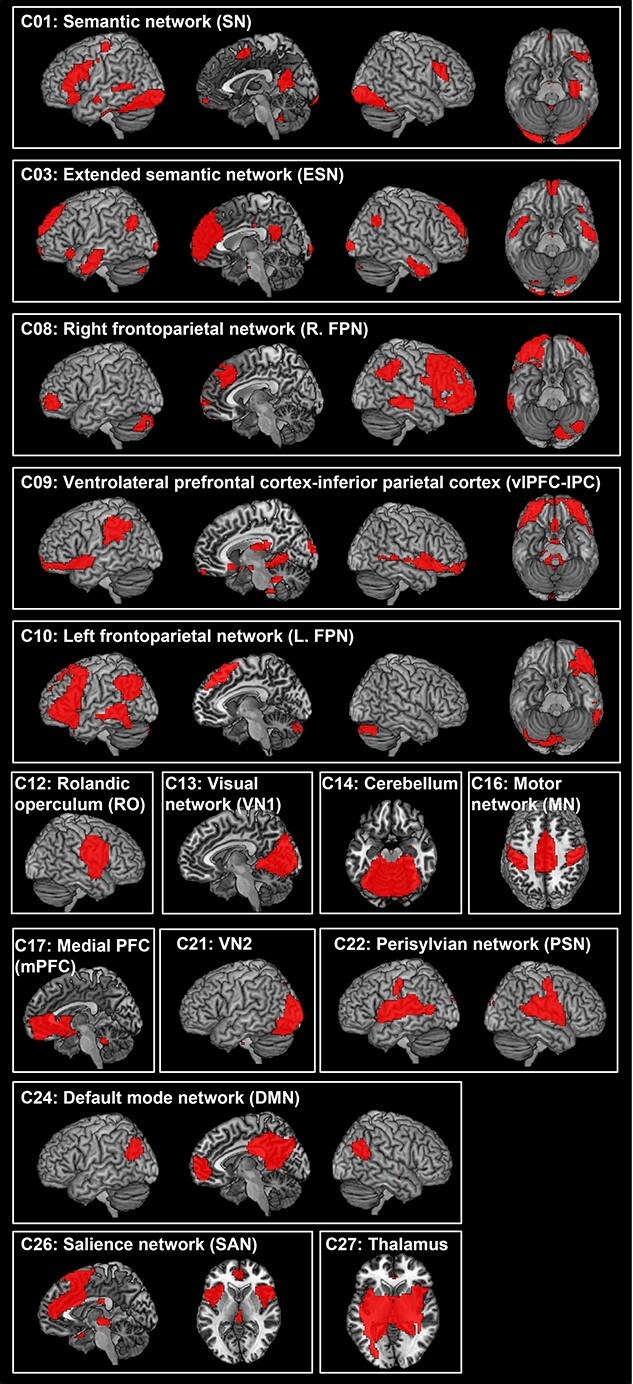
The ICA results. Statistical threshold was set at z > 4. See Supplementary Table S1 for coordinates.

The ICA revealed 15 components showing the patterns of temporally coherent signal within the brain. We refer to these components as brain networks and label them according to their spatial location (e.g. FPN) or previously described labels (e.g. DMN). Fig. 1 and Supplementary Table S1 summarize the results of these 15 components.

**Fig. 2:**
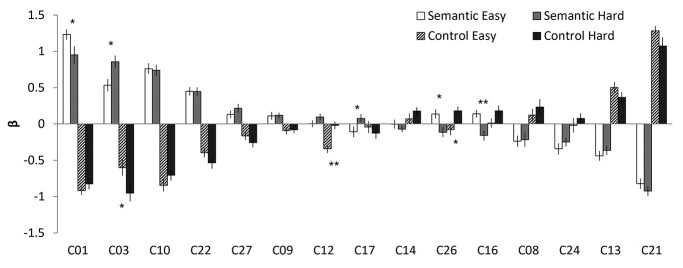
The results of temporal regression analysis. Bar graph shows the mean β weight for each condition against the baseline. White and grey bars represent SE and SH conditions, respectively. Patterned and black bars represent CE and CH conditions, respectively. Error bars indicate standard errors. Significant difference between easy and hard conditions is shown with ^*^  *P* < 0.05, ^**^  *P* < 0.01.

Figure 2 and Table 2 display the ICA results of temporal regression analysis, showing how the different networks were modulated by the task conditions. Semantic conditions (easy and hard) significantly activated C01 semantic network: C03 (extended semantic network: ESN), C10 (L.FPN), and C22 (perisylvian network: PSN) and suppressed C13 (visual network 1: VN1), C21 (VN2), and C24 (DMN). C27 (thalamus) was significantly activated by the hard semantic condition only. In contrast, both control conditions significantly activated C13 and C21 and suppressed the networks activated by semantic processing (C01, C06, C10, and C22). The easy control condition inhibited C12 (rolandic operculum: RO), whereas the hard condition activated C26 (salience network: SAN) and suppressed C27. The ICA results of spatial regression analysis are summarized in Supplementary Table S2.

**Table 2:** The result of ICA temporal regression. Bold indicates the significant results from one-sample *t*-tests. *P*_FDR-corrected_ < 0.005.

	SE		SH		CE		CH	
IC	T-value	*P*	T-value	*P*	T-value	*P*	T-value	*P*
C01 (SN)	**17.06**	**0.0000**	**8.07**	**0.0000**	**−15.15**	**0.0000**	**−10.95**	**0.0000**
C03 (ESN)	**6.78**	**0.0000**	**8.78**	**0.0000**	**−5.98**	**0.0000**	**−7.12**	**0.0000**
C08 (R.FPN)	-3.17	0.0044	-2.19	0.0393	1.53	0.1397	2.06	0.0515
C09 (vlPFC-IPC)	2.02	0.0554	3.11	0.0051	-1.89	0.0726	-1.60	0.1230
C10 (L.FPN)	**10.21**	**0.0000**	**9.37**	**0.0000**	**−9.88**	**0.0000**	**−9.10**	**0.0000**
C12 (RO)	0.05	0.9620	2.11	0.0469	**−5.84**	**0.0000**	-0.36	0.7209
C13 (VN1)	**−6.58**	**0.0000**	**−5.59**	**0.0000**	**7.18**	**0.0000**	**5.06**	**0.0000**
C14 (Cerebellum)	-0.02	0.9816	-1.79	0.0871	0.96	0.3470	2.89	0.0086
C16 (MN)	2.56	0.0177	-2.32	0.0302	0.20	0.8449	2.41	0.0246
C17 (mPFC)	-1.47	0.1570	1.35	0.1903	-0.51	0.6151	-1.57	0.1297
C21 (VN2)	**−11.71**	**0.0000**	**−14.15**	**0.0000**	**19.46**	**0.0000**	**9.03**	**0.0000**
C22 (PSN)	**7.88**	**0.0000**	**7.99**	**0.0000**	**−6.20**	**0.0000**	**−6.56**	**0.0000**
C24 (DMN)	**−4.46**	**0.0002**	**−4.15**	**0.0004**	-0.19	0.8485	1.12	0.2751
C26 (SAN)	2.22	0.0371	-1.67	0.1084	-1.03	0.3153	**3.34**	**0.0029**
C27 (Thalamus)	2.50	0.0205	**3.53**	**0.0019**	-2.89	0.0086	**−4.09**	**0.0005**

SN: semantic network; ESN: extended semantic network; R.FPN: right frontoparietal network; vlPFC-IPC: ventrolateral prefrontal cortex-inferior parietal cortex; L.FPN: left frontoparietal network; RO: rolandic operculum; VN: visual network; MN: motor network; mPFC: medial prefrontal cortex; PSN: perisylvian network; DMN: default mode network; SAN: salience network.

### Networks related to semantic processing

**Fig. 3:**
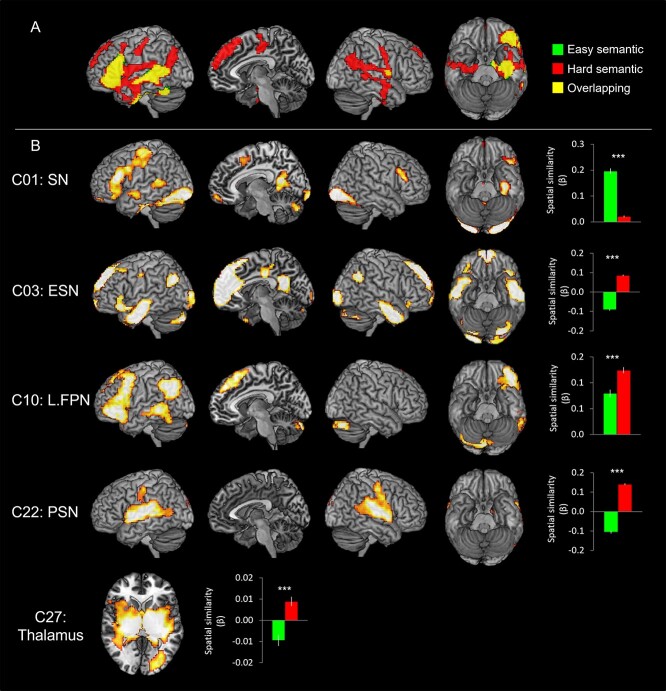
A) The GLM results of the semantic task. Green color indicates brain regions activated during the easy semantic processing and red color indicates brain areas during the hard semantic processing. Yellow color represents the overlapping regions. B) The ICA results of the semantic task. The temporal regression analysis identified that C01, C03, C10, C22, and C27 were significantly involved in the semantic task. The spatial regression analysis showed the spatial overlapping between the GLM results and the ICs. Green bars represent the spatial β weights for the easy condition and red bars for the hard condition. ^***^P_FDR- corrected_ < 0.001.

Figure 3 shows the brain activation maps from the GLM analysis and ICs related to semantic processing. The GLM revealed that the easy semantic condition evoked significant activation in the IFG, ventrolateral anterior temporal lobe (vATL), pMTG, fusiform gyrus (FG), and hippocampus in the left hemisphere as well as the inferior parietal lobe (IPL) and RO in the right hemisphere. The hard semantic condition induced more widespread activation in the same regions found in the easy semantic condition and additional activation in the bilateral AG, the right vATL, the mPFC, the middle cingulate cortex (MCC), and the pre/postcentral gyrus (Fig. 3A and Supplementary Table S3). In order to evaluate the difficulty effect in the key semantic regions, ROI analysis was performed in the vATL, IFG, and pMTG. The results showed that the hard semantic condition increased the activation in the left IFG and the right vATL significantly compared to the easy condition (*P* < 0.05). Also, there was a marginally significant increased activation in the left vATL and right pMTG during the hard semantic processing (*P* = 0.07) (Supplementary Fig. S2).

Different from the results of the GLM analysis, ICA revealed that 5 independent networks were involved in semantic processing, differently modulated by the task difficulty (Figs. 2 and 3).

C01 (SN) showed the biggest activation in semantic processing and deactivation in control processing. Similar to the GLM results, C01 consisted of the key semantic regions including the left vATL, left IFG (p. triangularis p. orbitalis, and p. opercularis), left pMTG, mPFC, SMA, bilateral FG, and precuneus. The temporal β weights of the network were analyzed by 2 × 2 ANOVA with task (semantic vs. control) and difficulty (easy vs. hard), and the results showed a significant main effect of task (*F*_1, 22_ = 216.01, *P* < 0.001), difficulty (*F*_1, 22_ = 6.59, *P* < 0.05), and an interaction (*F*_1, 22_ = 4.73, *P* < 0.05). Post hoc paired *t*-tests revealed that C01 was significantly more involved in the easy semantic condition compared to the hard condition (*P* < 0.05) (Fig. 2). Consistent to the results of temporal regression, spatial regression analysis demonstrated that the spatial overlapping was significantly greater between C01 and the easy semantic GLM results than the hard semantic results (Fig. 3B).

In contrast to C01, C03 (ESN) showed greater temporal and spatial engagement in the hard semantic condition compared to the easy condition. C03 included the bilateral ATL, left IFG (p. triangularis and p. orbitalis), AG, mPFC, superior frontal gyrus (SFG), ACC/MCC/posterior cingulate cortex (PCC), and visual cortex. 2 × 2 ANOVA with task and difficulty on the temporal β weights demonstrated a significant main effect of task (*F*_1, 22_ = 141.66, *P* < 0.001) and an interaction (*F*_1, 22_ = 4.87, *P* < 0.05). Post hoc paired *t*-tests revealed that C03 showed significantly greater involvement in the hard semantic condition (*P* < 0.05) (Fig. 2). Also, the spatial β weights revealed that C03 was more involved in the hard semantic condition, showing greater overlapping between the IC and GLM results derived from the hard processing (Fig. 3).

C10 (L.FPN) includes the left lateral prefrontal cortex, left IPL, and left pMTG and was activated for semantic processing, regardless of task difficulty. L.FPN is active for a wide range of tasks and thought to be involved in modulating cognitive control (Zanto and Gazzaley  2013). However, we found that C10 was activated by the semantic task and suppressed by the control task (Fig. 2). The temporal β weights ANOVA revealed a significant main effect of task (*F*_1, 22_ = 219.23, *P* < 0.001) and difficulty (*F*_1, 22_ = 5.06, *P* < 0.05). Contrary to the temporal regression, the spatial regression showed that C10 was overlapped with the brain activation map of the hard semantic condition more than that of the easy semantic condition (Fig. 3B).

C22 (PSN) showed significant semantic-related activity regardless of task difficulty (Fig. 2). The PSN includes the bilateral superior temporal gyrus, insular, RO, and the right supramarginal gyrus (SMG). The temporal β weights ANOVA showed a significant main effect of task (*F*_1, 22_ = 121.27, *P* < 0.001) and difficulty (*F*_1, 22_ = 8.23, *P* < 0.01). Although the temporal regression did not show a significant difference between easy and hard semantic condition, the spatial regression demonstrated that C22 was significantly overlapped with the GLM brain activation map from the hard semantic processing but not with the GLM result of the easy processing (Fig. 3B).

C27 (thalamus) consists of the thalamus, putamen, and pallidum. The temporal β weights ANOVA showed a significant main effect of task (*F*_1, 22_ = 25.21, *P* < 0.001). The temporal regression results revealed that the thalamus was significantly involved in the hard semantic condition (Fig. 2). The spatial β weights analysis showed that C27 was negatively associated with the easy semantic condition, whereas positively related with the hard semantic processing (Fig. 3B).

Several other components either failed to activate for semantic processing or showed deactivations (Fig. 2). C17 (mPFC) showed a significant differential involvement in semantic processing according to the task difficulty. C17 was inactive for the easy semantic condition, whereas active for the hard condition. In contrary, C16 (motor network: MN) and C26 (SAN) showed the opposite pattern, active for the easy condition and inactive for the hard condition.

### Networks related to visuospatial processing

**Fig. 4:**
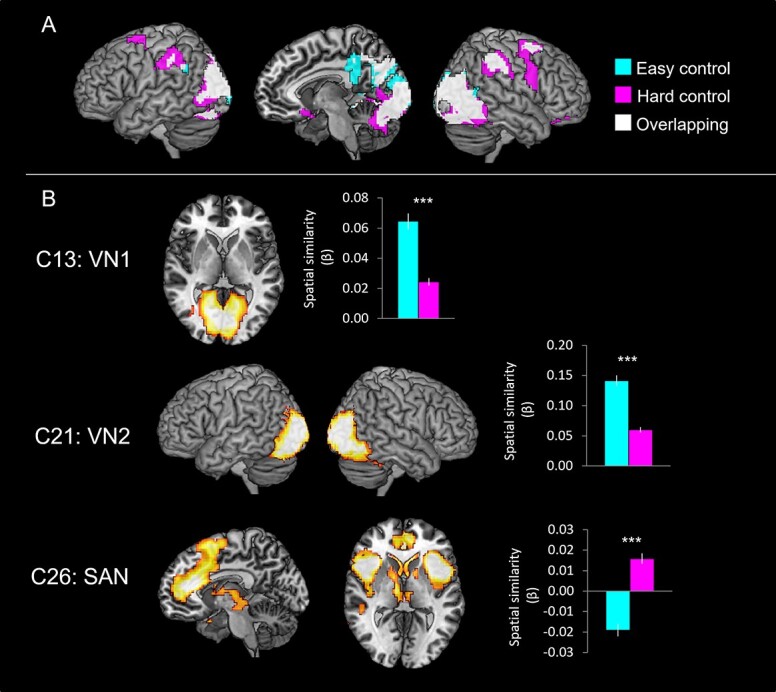
A) The GLM results of the control task. Cyan color indicates brain regions activated during the easy control processing and pink color indicates brain areas during the hard control processing. White color represents the overlapping regions. B) The ICA results of the control task. The temporal regression analysis identified that C13, C21, and C26 were significantly involved in the control task. The spatial regression analysis showed the spatial overlapping between the GLM results and the ICs. Cyan bars represent the spatial β weights for the easy condition and pink bars for the hard condition. ^***^P_FDR- corrected_ < 0.001.

Figure 4 shows the brain activation maps from the GLM analysis and ICs related to visuospatial processing (pattern matching). The GLM demonstrated that the easy control condition induced significant activation in the SFG, precuneus, and the bilateral visual cortices including the superior/MOG, FG, calcarine gyrus, and lingual gyrus. The hard condition also evoked the activation in the same regions found in the easy condition and in the middle frontal gyrus (MFG), insular, right IFG, and right middle orbital gyrus (Fig. 4A and Supplementary Table S3).

ICA revealed that 3 independent networks were involved in pattern matching processing and were differentially modulated by task difficulty (Figs. 2 and 4B). C13 (VN1) including the calcarine gyrus and lingual gyrus was significantly activated by the control task. 2 × 2 ANOVA with task and difficulty on the temporal β weights demonstrated a significant main effect of task (*F*_1, 22_ = 64.86, *P* < 0.001) only. The spatial regression showed that C13 was significantly overlapping with the brain activation map of the easy condition than the hard condition (Fig. 4B). Similar to C13, C21 (VN2) was significantly associated with the pattern matching processing during the easy condition. C21 consisting of the middle/inferior occipital gyrus and FG showed a significant main effect of task (*F*_1, 22_ = 327.33, *P* < 0.001) and difficulty (*F*_1, 22_ = 16.54, *P* < 0.001) in the temporal regression analysis (Fig. 2). There was no significant interaction. The spatial regression revealed that C21 also was involved in the easy condition more than the hard condition (Fig. 4B). In contrast to the VNs, C26 (SAN) showed significant control task relate activity only for the hard condition (Fig. 2). C26 includes the ACC, SMA, MFG, insular, and caudate nucleus and is thought to be critical for detecting behaviorally relevant stimuli and for coordinating the brain’s neural resources in response to these stimuli (Uddin 2017). The temporal β weights of C26 revealed a significant interaction effect between task and difficulty (*F*_1, 22_ = 8.59, *P* < 0.01). Post hoc paired *t*-tests revealed that C26 was significantly greater involvement in the hard control condition (Fig. 2). The spatial β weights analysis showed that C26 was negatively associated with the easy control condition, whereas positively related with the hard visual processing (Fig. 4B).

### Networks related to the interaction between task and difficulty

**Fig. 5:**
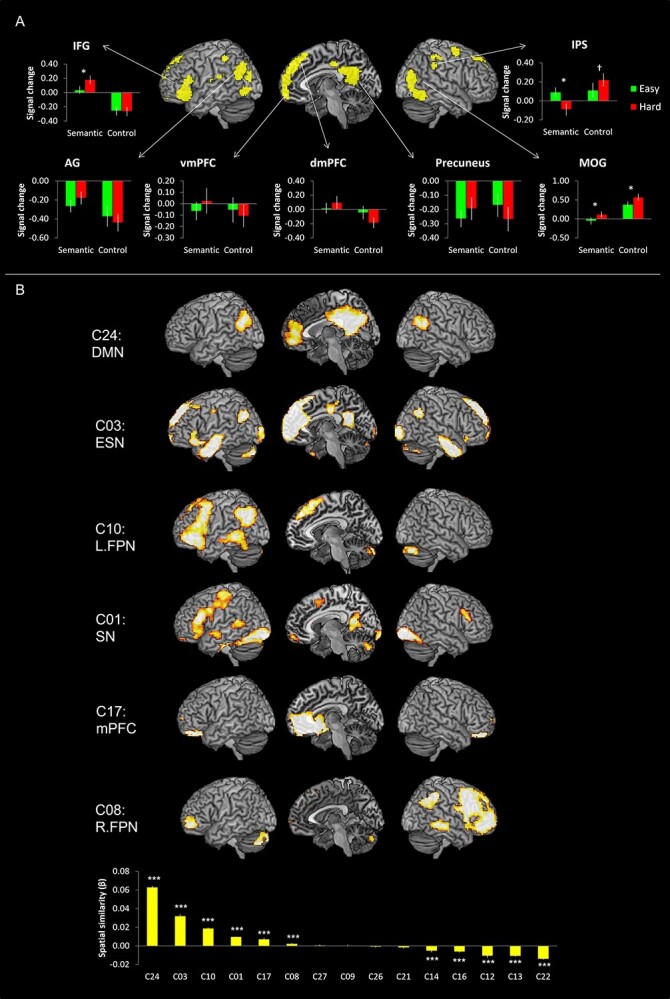
A) The GLM results of the interaction between task and difficulty. Green bars represent regional signal changes for the easy condition and red bars for the hard condition. B) The spatial regression results. The spatial regression analysis identified that C01, C03, C08, C10, C17, and C24 were significantly associated with the brain activation map of the interaction between task and difficulty. ^*^P < 0.05, ^†^*P* = 0.06, ^***^P_FDR- corrected_ < 0.001.

A significant interaction between task and difficulty was observed in the activation of the left IFG, left AG, mPFC, precuneus, right IPS, right MOG, and right inferior temporal gyrus (Fig. 5A and Supplementary Table S1). Specifically, the left IFG and vmPFC/dmPFC showed the increased activation only when the semantic task was hard. The right IPS and MOG were activated by the control task and more activated during the hard control condition.

In order to evaluate which ICs were overlapping with the brain map for the interaction between task and difficulty, we performed a spatial regression analysis. The results revealed that 6 ICs were significantly associated with the GLM interaction results (negative value means dissimilarity between the spatial maps so we did not report, one-sample *t*-test, *P*_FDR-corrected_ < 0.001) (Fig. 5B Bottom): specifically, C24 (DMN), C03 (ESN), C10 (L.FPN), C01 (SN), C17 (mPFC), and C08 (R.FPN). Of these networks, C01, C03, and C10 were found to have been positively engaged by semantic processing in the previous analyses (Figs. 2 and 3). In contrast, activation in C08 (R.FPN) was modulated by difficulty for the control visual processing task, specifically (Fig. 2 and Table 2).

### Functional network connectivity

**Fig. 6:**
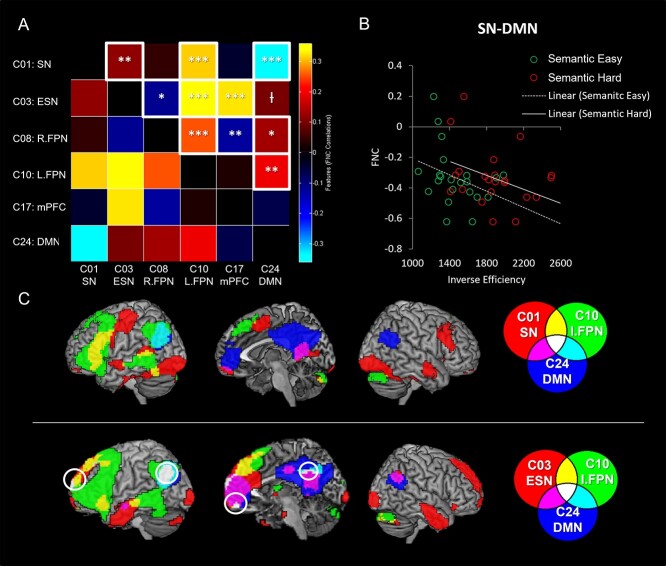
A) The results of FNC between the networks involved in task and difficulty. Red colors indicate positive coupling and blue colors indicate negative coupling. White box represents a significant FNC between networks (*P*_FDR-corrected_ < 0.05). B) The relationship between the FNC (SN-DMN) and semantic task performance (IE score: RT/accuracy × 100). Green and red circles represent individual performance during easy and hard semantic conditions, respectively. C) The overlapping between domain-specific (semantic processing) and domain-general networks. ^*^P_FDR-corrected_ < 0.05, ^**^P_FDR-corrected_ < 0.01, ^***^P_FDR-corrected_ < 0.001, ^†^*P*_FDR-corrected_ = 0.07.

We examined the internetwork connectivity between the ICs that showed a significant involvement in the interaction of task and difficulty (Fig. 6). Correlation analysis was performed between the time-courses of the 6 ICs (SN, ESN, R.FPN, L.FPN, mPFC, and DMN). C01 (SN) was significantly correlated with C03 (ESN) and C10 (L.FPN) yet was anticorrelated with C24 (DMN). C03 (ESN) was decoupled with C08 (R.FPN) and coupled with C10 (L.FPN) and C17 (mPFC). In addition, C03 (ESN) was marginally associated with the C24 (DMN) (*P*_FDR-corrected_ = 0.07). C08 (R.FPN) was positively correlated with C10 (L.FPN) and C24 (DMN), but negatively correlated with C17 (mPFC). C10 (L.FPN) was positively correlated with C24 (DMN). The results are displayed in Fig. 6A.

To explore the relationship between these FNC results and semantic task performance, we conducted an additional correlation analysis between the strength of the FNC and semantic performance. To combine accuracy and RT into a unitary measure of semantic performance, we computed the inverse efficiency (IE) score by dividing RT by accuracy. We found that the internetwork connectivity between the SN and DMN was significantly correlated with semantic task performance (Fig. 6B). This reflected the fact that participants with the least efficient semantic performance exhibited the largest anticorrelation between the 2 networks, for both levels of task difficulty (easy: *r* = −0.54, *P*_FDR-corrected_ < 0.05; hard: *r* = −0.47, *P*_FDR-corrected_ = 0.07).

### Overlapping networks

The ICA decomposes the fMRI data into multiple components with independent sources of variance. Although these components are spatiotemporally independent, they can partially overlap spatially. Figure 6C shows the overlapping regions between domain-specific (semantic processing) and domain-general networks. The SN overlapped with the L.FPN in the left IFG and pMTG and with the DMN in the mPFC and PCC. The left AG was common for the L.FPN and the DMN. The ESN overlapped with the L.PFN in the ventral IFG, dmPFC and MTG and with the DMN in the mPFC, PCC, and the right AG. The left AG, vmPFC, dmPFC, and PCC were common regions for all 3 networks.

## Discussion

This study investigated the neural dynamics of the functional brain networks that support semantic cognition, a key component of human higher cognition. Specifically, we manipulated task difficulty to explore task-related reconfiguration of brain networks during semantic processing. Using ICA, we found that semantic cognition required the cooperation of multiple brain networks, subserving distinctive cognitive processes spanning semantic and domain-general functions. Our task difficulty manipulation demonstrated the segregation and integration between domain-specific (semantic) networks and domain-general systems including the FPN and DMN. First, we found that 2 semantic networks (SN and ESN) exhibited differential sensitivity. The core SN (including ATL, IFG, and pMTG) was strongly engaged by both the easier and harder semantic task. The ESN (including bilateral ATL, ventral IFG, mPFC, AG, and precuneus) was additionally recruited for the more demanding version of the semantic task. Together, the SN and ESN reflect the widely distributed, large-scale neural systems implicated in semantic cognition by other researchers (Binder et al. 2009; Binder and Desai 2011; Lambon Ralph et al. 2017; Jackson 2021). Importantly, our findings support the proposal that semantic cognition is founded on a flexible, dynamic system, revealing its resilience to varying task demands, neurostimulation, and brain damage (Jung and Lambon Ralph 2016; Rice et al. 2018b; Stefaniak et al. 2020; Jung et al. 2021). Second, the functional contributions of various domain-general networks were segregated by the task and difficulty manipulations: L.FPN was engaged in semantic processing, whereas R.FPN was associated with visuospatial processing. In line with past explorations (Pexman et al. 2007; Geranmayeh et al. 2012; Humphreys et al. 2015; Humphreys and Lambon Ralph 2017; Humphreys et al. 2022), the core DMN showed a distinct disinterest in semantic processing showing deactivation for both versions of the semantic tasks, and a significant anticorrelation with the core SN. In turn, we found that the degree of anticorrelation between the SN and DMN was related to the varying semantic performance across participants; those with the least efficient performance exhibited the greatest anticorrelation. Overall, our results revealed the flexible, neural dynamics of semantic cognition as implemented by large-scale reconfiguration and integration of higher cognitive brain networks (Bressler and Kelso 2001; Cohen and D'Esposito 2016).

The CSC framework proposes that semantic cognition requires 2 interactive components: semantic representation and control (Lambon Ralph et al. 2017). Past patient, repetitive transcranial magnetic stimulation (rTMS) and fMRI studies implicate 2 networks for semantic control: a domain-specific set of ventral IPFC and posterior MTG regions as well as the more domain-general, multi-demand DLPFC and IPS areas (Jefferies and Lambon Ralph 2006; Jefferies et al. 2007; Hoffman et al. 2010; Jefferies 2013; Noonan et al. 2013; Jackson 2021). These 2 networks fall within the FPN identified in the current study. Indeed, areas within the FPN have been identified for executive control (Duncan and Owen 2000; Fedorenko et al. 2013) and play an important role in semantic retrieval (Badre et al. 2005; Nagel et al. 2008). Specifically, recent computational models have shown how this executive system might modulate and constrain the activation of semantic representations (Hoffman et al.  2018; Jackson et al. 2021). Our results revealed that the FPN was actively engaged in semantic processing and also interacted with 2 semantic networks, consistent with past patient and fMRI explorations (Jefferies and Lambon Ralph 2006; Jefferies et al. 2007; Noonan et al. 2013; Chiou et al. 2018; Jung et al. 2021). Our network-level analyses were able to go further. ICA decomposed the FPN into 2 networks, showing differential task modulations of the FPN: L.FPN for semantic processing and R.FPN for nonsemantic processing. Furthermore, L.FPN was positively synchronized with 2 semantic networks, whereas R.FPN was decoupled with the ESN. This result might reflect that our language-based semantic task induced more involvement of the left-lateralized FPN. Our findings support the recent proposal that the FPN can be reorganized, depending on specific task demands, by fractionizing itself or recruiting additional regions (Parlatini et al. 2017; Camilleri et al. 2018).

### The importance of dynamic large-scale networks for resilient neural systems

Recently, we proposed a new neurocognitive principle, “variable neuro-displacement,” to describe how intrinsic, dynamic brain mechanisms could support both response to varying performance demands and provide a basis for resilient cognition (Stefaniak et al. 2020). A key idea at the heart of this proposal is that the neural basis of cognition not only needs to support all aspects of higher cognition but also be able to respond to natural variations in performance demand. In addition, given that the brain is a very metabolically expensive organ, cognitive performance will always need to be titrated against energy expenditure. Accordingly, functional neural systems will be downregulated to save energy under normal levels of performance demand but have extra capacity that can be engaged when needed. As a result, the dynamic system provides an intrinsic, generic mechanism for resilience to a full range of situations including performance variations, transient brain inefficiency, or brain damage (Stefaniak et al. 2020). Previously, we tested this principle in the semantic domain by manipulating performance demand (Jung et al. 2021), after rTMS (Jung and Lambon Ralph 2016) and after selective damage (ATL resection for treatment of temporal lobe epilepsy) (Rice et al. 2018b). There was strong convergence across these studies, with the results demonstrating that demanding/disrupted semantic processing evoked upregulation in the left-lateralized SN and strengthened functional connectivity within the system. There was additional activation in the right ATL, and key regions in the FPN as well as increased functional connectivity between them. In this study, we replicated these findings and expanded them to the network level. Our ICA revealed 2 distinctive semantic networks: the left-lateralized SN (C01) and the ESN (C03). In line with the variable neuro-displacement hypothesis, when semantic demands are increased, the core SN (involved in all levels of semantic processing) is joined by the ESN, thus providing a network-level basis for resilient semantic cognition.

The bilateral semantic representation system has been observed in previous studies (Humphreys et al. 2015; Rice et al. 2015; Jung and Lambon Ralph 2016; Humphreys and Lambon Ralph 2017; Jung et al. 2021). For example, inhibition of this system using rTMS, triggered compensatory upregulation in the contralateral ATL to the stimulated ATL (Binney and Lambon Ralph 2014) and increased ATL-connectivity (Jung and Lambon Ralph 2016). Importantly, patients with bilateral ATL atrophy suffer from substantial semantic impairments, potentially due to the destruction of the resilience of the system, whereas patients and nonhuman primates with unilateral ATL damage showed relatively sustained semantic function (Kluver and Bucy 1939; Terzian and Ore 1955; Lambon Ralph et al. 2010a; Rice et al. 2018a; Ding et al. 2020). Exploration of the neural basis of semantic function in patients with unilateral ATL resection (Rice et al. 2018b) observed similar compensatory mechanisms to those found in rTMS studies and our current ICA network-level results, including upregulation in the contralateral regions, increased connectivity within the system, and engagement of the FPN. These findings imply that the bilateral nature of ATL is crucial for a well-engineered, resilient semantic representation, making the system robust to damage or task demands with greater capacity for experience-dependent neural plasticity (Warren et al. 2009; Schapiro et al. 2013; Jung and Lambon Ralph 2016; Chang and Lambon Ralph 2020; Jung et al. 2021). In addition, the reconfiguration of the FPN and its intercorrelations with the SNs may contribute to the resilience of semantic cognition by increasing its involvement for demanding semantic processing (Jung et al. 2021).

### Implications for the relationship between SN and DMN

Our results might have significant implications for interpretation of the relationship between the SN and DMN. Our data demonstrated that the core SN and DMN seem to be 2 distinctive neural systems not only in terms of ICA spatiotemporal separation but also functionally. Specifically, the SN (C01) was activated, whereas DMN (C24) was deactivated during semantic processing (Fig. 2). Furthermore, we found that the SN was decoupled with DMN during semantic processing (Fig. 6A). These results fit with those from recent large-scale fMRI meta-analysis and direct multi-task fMRI comparisons (Humphreys et al. 2015; Humphreys and Lambon Ralph 2015, 2017; Humphreys et al. 2020; Humphreys et al. 2022), which have shown a stark contrast between the core SN regions including ATL (that show very strong engagement) and the AG alongside the core midline DMN areas (that show deactivation for semantic and other tasks). Indeed, in contrast to the deactivation during semantic processing, the AG and core DMN areas are positively activated during episodic retrieval instead (Wagner et al. 2005; Rugg and Vilberg 2013; Wagner et al. 2015; Rugg and King 2018; Humphreys et al. 2021; Humphreys et al. 2022).

A second repeatedly replicated yet often overlooked feature of the DMN is that its deactivation across many tasks (other than episodic memory) is not constant. Instead, the level of deactivation is correlated with task/item difficulty (Laurienti et al. 2002; Fransson 2006; McKiernan et al. 2006; Mason et al. 2007; Mayer et al. 2010). This fact aligns with another finding from the current study and others from the literature. First, in the current study, we found that the degree of decoupling between the SN and DMN was related to semantic task performance: individuals with the greatest decoupling exhibited the least efficient semantic processing. This result could reflect the same difficulty-related variation of deactivation: i.e. like the variation of deactivation induced by harder tasks or items, the DMN shows enhanced deactivation for participants who find a specific task harder. This same variation in deactivation of the DMN is also important for explaining other key results in the literature: (i) the apparent engagement of AG and other DMN in fMRI contrasts such as words > nonwords or concrete > abstract concepts (Binder et al. 2009; Wang et al. 2010) may well reflect a contrast of easy > hard conditions. Indeed, the AG and DMN appear in fMRI maps when contrasting easy > hard in entirely nonsemantic, nonverbal tasks (Humphreys and Lambon Ralph 2017), or when the difficulty for words versus nonwords, or concrete versus abstract decisions is reversed (Pexman et al. 2007). Thirdly and relevant to the network-level focus of the current study, in simple fMRI correlations/“functional connectivity” analyses (e.g. in resting-state fMRI) the ATL and DMN areas are often coupled, yet in the current ICA analyses (and parallel studies; Geranmayeh et al. 2012; Jackson et al. 2019) they are pulled apart. Indeed, Buckner et al. (2008) note that the ATL areas are an inconsistent member of the extended DMN, whereas the AG and midline areas are more constant features of the DMN. The coupling of the networks in simple functional connectivity analyses might also reflect partially shared deactivation profiles: thus whilst the ATL and related regions activate for semantic tasks, and the AG + DMN for episodic retrieval, they both deactivate commonly in other task-active conditions (e.g. auditory or visual perceptual decisions; Humphreys et al. 2015). Thus, their partial simple correlation could simply reflect the regions shared disinterest in a range of cognitive and perceptual activities.

Interestingly, we found that the inferior parietal lobule including AG is a common region for the ESN, FPN, and DMN. This finding can be explained by a unifying model of parietal function, the Parietal Unified Connectivity-biased Computation (PUCC) (Humphreys and Lambon Ralph 2015). The PUCC model assumes that the local computation of lateral parietal cortex (LPC) is online and multisensory buffering across modalities, which is critical to process time-extended cognition such as episodic memory or sentence processing (Hasson et al. 2008; Lerner et al. 2011). The second assumption is that although the local computation may be constant across the LPC, there are graded subregions showing task-specific effects determined by its long-range connections (Uddin et al. 2010; Cloutman et al. 2013; Fedorenko et al. 2013; Humphreys et al. 2022). In line with these proposals, we showed distinctive functional connectivity patterns across the subregions within the LPC: the dorsal LPC (IPS/dorsal AG) formed the FPN, anterior AG was connected with frontotemporal language network, middle AG formed the DMN, and posterior AG was connected with visuospatial network (Humphreys and Lambon Ralph 2017; Humphreys et al. 2020; Humphreys et al. 2022).

### Other neural networks involved in semantic processing

We found that an mPFC network (C17) was activated for demanding semantic processing. Recent studies reported that mPFC showed a graded change in structural and functional connectivity from DMN to ATL such that dmPFC was connected to the DMN, whereas vmPFC was linked to the SN (Jackson et al. 2020; Jung et al. 2022). As vmPFC has been associated with economic value judgment (Padoa-Schioppa and Assad 2006) and cognitive flexibility (Jobson et al. 2021), this region might contribute to semantic assessment of a context-specific item when the task was challenging (Noonan et al. 2013; Stalnaker et al. 2015). Alternatively, the mPFC and precuneus have been identified as brain network hubs for integrative processes and communications between functional systems (van den Heuvel and Sporns 2013). Thus, the recruitment of the mPFC during semantic processing might reflect a general mental resource for challenging situations.

We found the PSN (C22) was positively engaged in semantic processing. The PSN is a bilateral system including the superior temporal gyrus, insular, RO, and SMG. Given that the perisylvian region is known to support both receptive and expressive aspects of language (Hickok and Poeppel 2007), and damage leads to different forms of classical aphasia (Geschwind 1972; Ueno et al. 2011), then it seems most likely that PSN was recruited because the semantic task was verbal.

In addition to cortical networks, the thalamus network (C27) also participated in semantic processing for the demanding condition. This network includes thalamus, putamen, and pallidum. This system has been considered as a station relaying all incoming information from outside world to the cortex (McCormick and Bal 1994). Several studies have suggested that both the thalamus and the putamen are a part of the multi-demand system that links different regions via cortico-striatal-thalamic circuits (Alexander et al. 1986; Bonelli and Cummings 2007; Camilleri et al. 2018). Evidence for cortico-thalamic language processing has also been provided by a study with simultaneous depth and scalp recordings in the context of deep brain stimulation (Wahl et al. 2008). During syntactic processing, language-related potentials (LRP) were identified in the ventrolateral thalamus. During semantic processing, both cortical and thalamic LRPs appeared. Taken together, our data suggest that the thalamus system may be recruited for semantic processing in parallel to domain-general processing, operated through cortico-striatal-thalamic circuits (Ullman 2006).

## Conclusion

Our findings provide insights about the large-scale, task-related reconfiguration of brain networks in semantic cognition. In particular, our data showed segregation and integration of multiple brain networks not only in line with which task is being performance but also with increasing cognitive complexity.

## Funding

This research was supported by a Beacon Anne McLaren Research Fellowship (University of Nottingham) to JJ and an Advanced European Research Council Award (GAP: 670428—BRAIN2MIND_NEUROCOMP), Medical Research Council Programme Grant (MR/R023883/1), and Intramural Funding (MC_UU_00005/18) to MALR.

*Conflict of interest statement*. The authors declare no conflict of interest.

## Supplementary Material

Supplementary_Information_bhac190

## References

[ref1] Alexander GE, DeLong MR, Strick PL. Parallel organization of functionally segregated circuits linking basal ganglia and cortex. Annu Rev Neurosci. 1986:9:357–381. 3085570 10.1146/annurev.ne.09.030186.002041

[ref2] Ashburner J . A fast diffeomorphic image registration algorithm. NeuroImage. 2007:38:95–113. 17761438 10.1016/j.neuroimage.2007.07.007

[ref3] Badre D, Wagner AD. Semantic retrieval, mnemonic control, and prefrontal cortex. Behav Cogn Neurosci Rev. 2002:1:206–218. 17715593 10.1177/1534582302001003002

[ref4] Badre D, Poldrack RA, Pare-Blagoev EJ, Insler RZ, Wagner AD. Dissociable controlled retrieval and generalized selection mechanisms in ventrolateral prefrontal cortex. Neuron. 2005:47:907–918. 16157284 10.1016/j.neuron.2005.07.023

[ref5] Bell AJ, Sejnowski TJ. An information maximization approach to blind separation and blind deconvolution. Neural Comput. 1995:7:1129–1159. 7584893 10.1162/neco.1995.7.6.1129

[ref6] Binder JR, Desai RH. The neurobiology of semantic memory. Trends Cogn Sci. 2011:15:527–536. 22001867 10.1016/j.tics.2011.10.001 PMC3350748

[ref7] Binder JR, Frost JA, Hammeke TA, Bellgowan PS, Rao SM, Cox RW. Conceptual processing during the conscious resting state. A functional MRI study. J Cogn Neurosci. 1999:11:80–95. 9950716 10.1162/089892999563265

[ref8] Binder JR, Desai RH, Graves WW, Conant LL. Where is the semantic system? A critical review and meta-analysis of 120 functional neuroimaging studies. Cereb Cortex. 2009:19:2767–2796. 19329570 10.1093/cercor/bhp055 PMC2774390

[ref9] Binder JR, Conant LL, Humphries CJ, Fernandino L, Simons SB, Aguilar M, Desai RH. Toward a brain-based componential semantic representation. Cogn Neuropsychol. 2016:33:130–174. 27310469 10.1080/02643294.2016.1147426

[ref10] Binney RJ, Lambon Ralph MA. Using a combination of fMRI and anterior temporal lobe rTMS to measure intrinsic and induced activation changes across the semantic cognition network. Neuropsychologia. 2015:76:170–181. 25448851 10.1016/j.neuropsychologia.2014.11.009 PMC4582802

[ref11] Binney RJ, Embleton KV, Jefferies E, Parker GJ, Ralph MA. The ventral and inferolateral aspects of the anterior temporal lobe are crucial in semantic memory: evidence from a novel direct comparison of distortion-corrected fMRI, rTMS, and semantic dementia. Cereb Cortex. 2010:20:2728–2738. 20190005 10.1093/cercor/bhq019

[ref12] Bonelli RM, Cummings JL. Frontal-subcortical circuitry and behavior. Dialogues Clin Neurosci. 2007:9:141–151. 17726913 10.31887/DCNS.2007.9.2/rbonelli PMC3181854

[ref13] Bozeat S, Lambon Ralph MA, Patterson K, Garrard P, Hodges JR. Non-verbal semantic impairment in semantic dementia. Neuropsychologia. 2000:38:1207–1215. 10865096 10.1016/s0028-3932(00)00034-8

[ref14] Bressler SL, Kelso JA. Cortical coordination dynamics and cognition. Trends Cogn Sci. 2001:5:26–36. 11164733 10.1016/s1364-6613(00)01564-3

[ref15] Buckner RL, Andrews-Hanna JR, Schacter DL. The brain's default network: anatomy, function, and relevance to disease. Ann N Y Acad Sci. 2008:1124:1–38. 18400922 10.1196/annals.1440.011

[ref16] Calhoun VD, Adali T, McGinty VB, Pekar JJ, Watson TD, Pearlson GD. fMRI activation in a visual-perception task: network of areas detected using the general linear model and independent components analysis. NeuroImage. 2001a:14:1080–1088. 11697939 10.1006/nimg.2001.0921

[ref17] Calhoun VD, Adali T, Pearlson GD, Pekar JJ. A method for making group inferences from functional MRI data using independent component analysis. Hum Brain Mapp. 2001b:14:140–151. 11559959 10.1002/hbm.1048 PMC6871952

[ref18] Camilleri JA, Muller VI, Fox P, Laird AR, Hoffstaedter F, Kalenscher T, Eickhoff SB. Definition and characterization of an extended multiple-demand network. NeuroImage. 2018:165:138–147. 29030105 10.1016/j.neuroimage.2017.10.020 PMC5732056

[ref19] Chang YN, Lambon Ralph MA. A unified neurocomputational bilateral model of spoken language production in healthy participants and recovery in poststroke aphasia. Proc Natl Acad Sci U S A. 2020:117:32779–32790. 33273118 10.1073/pnas.2010193117 PMC7768768

[ref20] Chen Y, Shimotake A, Matsumoto R, Kunieda T, Kikuchi T, Miyamoto S, Fukuyama H, Takahashi R, Ikeda A, Lambon Ralph MA. The 'when' and 'where' of semantic coding in the anterior temporal lobe: temporal representational similarity analysis of electrocorticogram data. Cortex. 2016:79:1–13. 27085891 10.1016/j.cortex.2016.02.015 PMC4884671

[ref21] Chiou R, Humphreys GF, Jung J, Lambon Ralph MA. Controlled semantic cognition relies upon dynamic and flexible interactions between the executive 'semantic control' and hub-and-spoke 'semantic representation' systems. Cortex. 2018:103:100–116. 29604611 10.1016/j.cortex.2018.02.018 PMC6006425

[ref22] Cloutman LL, Binney RJ, Morris DM, Parker GJ, Lambon Ralph MA. Using in vivo probabilistic tractography to reveal two segregated dorsal 'language-cognitive' pathways in the human brain. Brain Lang. 2013:127:230–240. 23937853 10.1016/j.bandl.2013.06.005 PMC3842500

[ref23] Coccia M, Bartolini M, Luzzi S, Provinciali L, Ralph MA. Semantic memory is an amodal, dynamic system: evidence from the interaction of naming and object use in semantic dementia. Cogn Neuropsychol. 2004:21:513–527. 21038218 10.1080/02643290342000113

[ref24] Cohen JR, D'Esposito M. The segregation and integration of distinct brain networks and their relationship to cognition. J Neurosci. 2016:36:12083–12094. 27903719 10.1523/JNEUROSCI.2965-15.2016 PMC5148214

[ref25] Corbett F, Jefferies E, Ehsan S, Lambon Ralph MA. Different impairments of semantic cognition in semantic dementia and semantic aphasia: evidence from the non-verbal domain. Brain. 2009:132:2593–2608. 19506072 10.1093/brain/awp146 PMC2766180

[ref26] Ding J, Chen K, Liu H, Huang L, Chen Y, Lv Y, Yang Q, Guo Q, Han Z, Lambon Ralph MA. A unified neurocognitive model of semantics language social behaviour and face recognition in semantic dementia. Nat Commun. 2020:11:2595. 32444620 10.1038/s41467-020-16089-9 PMC7244491

[ref27] Domagalik A, Beldzik E, Fafrowicz M, Oginska H, Marek T. Neural networks related to pro-saccades and anti-saccades revealed by independent component analysis. NeuroImage. 2012:62:1325–1333. 22705376 10.1016/j.neuroimage.2012.06.006

[ref28] Duncan J . The multiple-demand (MD) system of the primate brain: mental programs for intelligent behaviour. Trends Cogn Sci. 2010:14:172–179. 20171926 10.1016/j.tics.2010.01.004

[ref29] Duncan J, Owen AM. Common regions of the human frontal lobe recruited by diverse cognitive demands. Trends Neurosci. 2000:23:475–483. 11006464 10.1016/s0166-2236(00)01633-7

[ref30] Fedorenko E, Duncan J, Kanwisher N. Language-selective and domain-general regions lie side by side within Broca's area. Curr Biol. 2012:22:2059–2062. 23063434 10.1016/j.cub.2012.09.011 PMC3494832

[ref31] Fedorenko E, Duncan J, Kanwisher N. Broad domain generality in focal regions of frontal and parietal cortex. Proc Natl Acad Sci U S A. 2013:110:16616–16621. 24062451 10.1073/pnas.1315235110 PMC3799302

[ref32] Fransson P . How default is the default mode of brain function? Further evidence from intrinsic BOLD signal fluctuations. Neuropsychologia. 2006:44:2836–2845. 16879844 10.1016/j.neuropsychologia.2006.06.017

[ref33] Geranmayeh F, Brownsett SL, Leech R, Beckmann CF, Woodhead Z, Wise RJ. The contribution of the inferior parietal cortex to spoken language production. Brain Lang. 2012:121:47–57. 22381402 10.1016/j.bandl.2012.02.005

[ref34] Geranmayeh F, Wise RJ, Mehta A, Leech R. Overlapping networks engaged during spoken language production and its cognitive control. J Neurosci. 2014:34:8728–8740. 24966373 10.1523/JNEUROSCI.0428-14.2014 PMC4069351

[ref35] Geschwind N . Language and the brain. Sci Am. 1972:226:76–83. 5014017 10.1038/scientificamerican0472-76

[ref36] Halai AD, Welbourne SR, Embleton K, Parkes LM. A comparison of dual gradient-echo and spin-echo fMRI of the inferior temporal lobe. Hum Brain Mapp. 2014:35:4118–4128. 24677506 10.1002/hbm.22463 PMC6869502

[ref37] Hasson U, Yang E, Vallines I, Heeger DJ, Rubin N. A hierarchy of temporal receptive windows in human cortex. J Neurosci. 2008:28:2539–2550. 18322098 10.1523/JNEUROSCI.5487-07.2008 PMC2556707

[ref39] Hickok G, Poeppel D. The cortical organization of speech processing. Nat Rev Neurosci. 2007:8:393–402. 17431404 10.1038/nrn2113

[ref40] Hoffman P, Jefferies E, Lambon Ralph MA. Ventrolateral prefrontal cortex plays an executive regulation role in comprehension of abstract words: convergent neuropsychological and repetitive TMS evidence. J Neurosci. 2010:30:15450–15456. 21084601 10.1523/JNEUROSCI.3783-10.2010 PMC6633672

[ref41] Hoffman P, McClelland JL, Lambon Ralph MA. Concepts, control, and context: a connectionist account of normal and disordered semantic cognition. Psychol Rev. 2018:125:293–328. 29733663 10.1037/rev0000094 PMC5937916

[ref42] Humphreys GF, Lambon Ralph MA. Fusion and fission of cognitive functions in the human parietal cortex. Cereb Cortex. 2015:25:3547–3560. 25205661 10.1093/cercor/bhu198 PMC4585503

[ref43] Humphreys GF, Lambon Ralph MA. Mapping domain-selective and counterpointed domain-general higher cognitive functions in the lateral parietal cortex: evidence from fMRI comparisons of difficulty-varying semantic versus visuo-spatial tasks, and functional connectivity analyses. Cereb Cortex. 2017:27:4199–4212. 28472382 10.1093/cercor/bhx107

[ref44] Humphreys GF, Hoffman P, Visser M, Binney RJ, Lambon Ralph MA. Establishing task- and modality-dependent dissociations between the semantic and default mode networks. Proc Natl Acad Sci U S A. 2015:112:7857–7862. 26056304 10.1073/pnas.1422760112 PMC4485123

[ref45] Humphreys GF, Jackson RL, Lambon Ralph MA. Overarching principles and dimensions of the functional organization in the inferior parietal cortex. Cereb Cortex. 2020:30:5639–5653. 32515783 10.1093/cercor/bhaa133 PMC7116231

[ref46] Humphreys GF, Lambon Ralph MA, Simons JS. A unifying account of angular gyrus contributions to episodic and semantic cognition. Trends Neurosci. 2021:44:452–463. 33612312 10.1016/j.tins.2021.01.006

[ref47] Humphreys GF, Jung J, Lambon Ralph MA. The convergence and divergence of episodic and semantic functions across lateral parietal cortex. Cereb Cortex. 2022:00:1–18. 10.1093/cercor/bhac044 PMC9753060 35196706

[ref48] Jackson RL . The neural correlates of semantic control revisited. NeuroImage. 2021:224:117444. 33059049 10.1016/j.neuroimage.2020.117444 PMC7779562

[ref49] Jackson RL, Hoffman P, Pobric G, Ralph MAL. The nature and neural correlates of semantic association versus conceptual similarity. Cereb Cortex. 2015:25:4319–4333. 25636912 10.1093/cercor/bhv003 PMC4816784

[ref50] Jackson RL, Hoffman P, Pobric G, Lambon Ralph MA. The semantic network at work and rest: differential connectivity of anterior temporal lobe subregions. J Neurosci. 2016:36:1490–1501. 26843633 10.1523/JNEUROSCI.2999-15.2016 PMC4737765

[ref51] Jackson RL, Cloutman LL, Lambon Ralph MA. Exploring distinct default mode and semantic networks using a systematic ICA approach. Cortex. 2019:113:279–297. 30716610 10.1016/j.cortex.2018.12.019 PMC6459395

[ref52] Jackson RL, Bajada CJ, Lambon Ralph MA, Cloutman LL. The graded change in connectivity across the ventromedial prefrontal cortex reveals distinct subregions. Cereb Cortex. 2020:30:165–180. 31329834 10.1093/cercor/bhz079 PMC7029692

[ref53] Jackson RL, Rogers TT, Lambon Ralph MA. Reverse-engineering the cortical architecture for controlled semantic cognition. Nat Hum Behav. 2021:5:774–786. 33462472 10.1038/s41562-020-01034-z PMC7611056

[ref54] Jafri MJ, Pearlson GD, Stevens M, Calhoun VD. A method for functional network connectivity among spatially independent resting-state components in schizophrenia. NeuroImage. 2008:39:1666–1681. 18082428 10.1016/j.neuroimage.2007.11.001 PMC3164840

[ref55] Jefferies E . The neural basis of semantic cognition: converging evidence from neuropsychology, neuroimaging and TMS. Cortex. 2013:49:611–625. 23260615 10.1016/j.cortex.2012.10.008

[ref56] Jefferies E, Lambon Ralph MA. Semantic impairment in stroke aphasia versus semantic dementia: a case-series comparison. Brain. 2006:129:2132–2147. 16815878 10.1093/brain/awl153

[ref57] Jefferies E, Baker SS, Doran M, Ralph MA. Refractory effects in stroke aphasia: a consequence of poor semantic control. Neuropsychologia. 2007:45:1065–1079. 17074373 10.1016/j.neuropsychologia.2006.09.009

[ref58] Jobson DD, Hase Y, Clarkson AN, Kalaria RN. The role of the medial prefrontal cortex in cognition, ageing and dementia. Brain Commun. 2021:3:fcab125. 34222873 10.1093/braincomms/fcab125 PMC8249104

[ref59] Jung J, Lambon Ralph MA. Mapping the dynamic network interactions underpinning cognition: a cTBS-fMRI study of the flexible adaptive neural system for semantics. Cereb Cortex. 2016:26:3580–3590. 27242027 10.1093/cercor/bhw149 PMC4961025

[ref60] Jung J, Visser M, Binney RJ, Lambon Ralph MA. Establishing the cognitive signature of human brain networks derived from structural and functional connectivity. Brain Struct Funct. 2018:223:4023–4038. 30120553 10.1007/s00429-018-1734-x PMC6267264

[ref61] Jung JY, Rice GE, Lambon Ralph MA. The neural bases of resilient semantic system: evidence of variable neuro-displacement in cognitive systems. Brain Struct Funct. 2021:226:1585–1599. 33877431 10.1007/s00429-021-02272-1 PMC8096767

[ref62] Jung J, Lambon Ralph MA, Jackson RL. Subregions of DLPFC display graded yet distinct structural and functional connectivity. J Neurosci. 2022:42:15:3241–3252. 10.1523/JNEUROSCI.1216-21.2022 PMC8994544 35232759

[ref63] Kim DI, Manoach DS, Mathalon DH, Turner JA, Mannell M, Brown GG, Ford JM, Gollub RL, White T, Wible C, et al. Dysregulation of working memory and default-mode networks in schizophrenia using independent component analysis, an fBIRN and MCIC study. Hum Brain Mapp. 2009:30:3795–3811. 19434601 10.1002/hbm.20807 PMC3058491

[ref64] Kluver H, Bucy PC. Preliminary analysis of functions of the temporal lobes in monkeys. Arch Neurol Psychiatr. 1939:42:979–1000. 10.1176/jnp.9.4.606 9447506

[ref65] Lambon Ralph MA, Cipolotti L, Manes F, Patterson K. Taking both sides: do unilateral anterior temporal lobe lesions disrupt semantic memory? Brain. 2010a:133:3243–3255. 20952378 10.1093/brain/awq264

[ref66] Lambon Ralph MA, Sage K, Jones RW, Mayberry EJ. Coherent concepts are computed in the anterior temporal lobes. P Natl Acad Sci USA. 2010b:107:2717–2722. 10.1073/pnas.0907307107 PMC2823909 20133780

[ref67] Lambon Ralph MA, Jefferies E, Patterson K, Rogers TT. The neural and computational bases of semantic cognition. Nat Rev Neurosci. 2017:18:42–55. 27881854 10.1038/nrn.2016.150

[ref68] Laurienti PJ, Burdette JH, Wallace MT, Yen YF, Field AS, Stein BE. Deactivation of sensory-specific cortex by cross-modal stimuli. J Cogn Neurosci. 2002:14:420–429. 11970801 10.1162/089892902317361930

[ref69] Lerner Y, Honey CJ, Silbert LJ, Hasson U. Topographic mapping of a hierarchy of temporal receptive windows using a narrated story. J Neurosci. 2011:31:2906–2915. 21414912 10.1523/JNEUROSCI.3684-10.2011 PMC3089381

[ref70] Martin A . GRAPES-grounding representations in action, perception, and emotion systems: how object properties and categories are represented in the human brain. Psychon Bull Rev. 2016:23:979–990. 25968087 10.3758/s13423-015-0842-3 PMC5111803

[ref71] Mason MF, Norton MI, Van Horn JD, Wegner DM, Grafton ST, Macrae CN. Wandering minds: the default network and stimulus-independent thought. Science. 2007:315:393–395. 17234951 10.1126/science.1131295 PMC1821121

[ref72] Mayer JS, Roebroeck A, Maurer K, Linden DE. Specialization in the default mode: task-induced brain deactivations dissociate between visual working memory and attention. Hum Brain Mapp. 2010:31:126–139. 19639552 10.1002/hbm.20850 PMC6870780

[ref73] McCormick DA, Bal T. Sensory gating mechanisms of the thalamus. Curr Opin Neurobiol. 1994:4:550–556. 7812144 10.1016/0959-4388(94)90056-6

[ref74] McKeown MJ, Sejnowski TJ. Independent component analysis of fMRI data: examining the assumptions. Hum Brain Mapp. 1998:6:368–372. 9788074 10.1002/(SICI)1097-0193(1998)6:5/6<368::AID-HBM7>3.0.CO;2-E PMC6873375

[ref75] McKiernan KA, D'Angelo BR, Kaufman JN, Binder JR. Interrupting the "stream of consciousness": an fMRI investigation. NeuroImage. 2006:29:1185–1191. 16269249 10.1016/j.neuroimage.2005.09.030 PMC1634934

[ref76] Nagel IE, Schumacher EH, Goebel R, D'Esposito M. Functional MRI investigation of verbal selection mechanisms in lateral prefrontal cortex. NeuroImage. 2008:43:801–807. 18692142 10.1016/j.neuroimage.2008.07.017 PMC2612124

[ref77] Noonan KA, Jefferies E, Visser M, Lambon Ralph MA. Going beyond inferior prefrontal involvement in semantic control: evidence for the additional contribution of dorsal angular gyrus and posterior middle temporal cortex. J Cogn Neurosci. 2013:25:1824–1850. 23859646 10.1162/jocn_a_00442

[ref78] Oldfield RC . The assessment and analysis of handedness: the Edinburgh inventory. Neuropsychologia. 1971:9:97–113. 5146491 10.1016/0028-3932(71)90067-4

[ref79] Padoa-Schioppa C, Assad JA. Neurons in the orbitofrontal cortex encode economic value. Nature. 2006:441:223–226. 16633341 10.1038/nature04676 PMC2630027

[ref80] Parlatini V, Radua J, Dell'Acqua F, Leslie A, Simmons A, Murphy DG, Catani M, Thiebaut de Schotten M. Functional segregation and integration within fronto-parietal networks. NeuroImage. 2017:146:367–375. 27639357 10.1016/j.neuroimage.2016.08.031 PMC5312783

[ref81] Patterson K, Nestor PJ, Rogers TT. Where do you know what you know? The representation of semantic knowledge in the human brain. Nat Rev Neurosci. 2007:8:976–987. 18026167 10.1038/nrn2277

[ref82] Peelen MV, Caramazza A. Conceptual object representations in human anterior temporal cortex. J Neurosci. 2012:32:15728–15736. 23136412 10.1523/JNEUROSCI.1953-12.2012 PMC6621609

[ref83] Pexman PM, Hargreaves IS, Edwards JD, Henry LC, Goodyear BG. Neural correlates of concreteness in semantic categorization. J Cogn Neurosci. 2007:19:1407–1419. 17651011 10.1162/jocn.2007.19.8.1407

[ref84] Raichle ME, MacLeod AM, Snyder AZ, Powers WJ, Gusnard DA, Shulman GL. A default mode of brain function. Proc Natl Acad Sci U S A. 2001:98:676–682. 11209064 10.1073/pnas.98.2.676 PMC14647

[ref85] Rice GE, Lambon Ralph MA, Hoffman P. The roles of left versus right anterior temporal lobes in conceptual knowledge: an ALE meta-analysis of 97 functional neuroimaging studies. Cereb Cortex. 2015:25:4374–4391. 25771223 10.1093/cercor/bhv024 PMC4816787

[ref86] Rice GE, Caswell H, Moore P, Hoffman P, Lambon Ralph MA. The roles of left versus right anterior temporal lobes in semantic memory: a neuropsychological comparison of postsurgical temporal lobe epilepsy patients. Cereb Cortex. 2018a:28:1487–1501. 29351584 10.1093/cercor/bhx362 PMC6093325

[ref87] Rice GE, Caswell H, Moore P, Lambon Ralph MA, Hoffman P. Revealing the dynamic modulations that underpin a resilient neural network for semantic cognition: an fMRI investigation in patients with anterior temporal lobe resection. Cereb Cortex. 2018b:28:3004–3016. 29878076 10.1093/cercor/bhy116 PMC6041810

[ref88] Rugg MD, King DR. Ventral lateral parietal cortex and episodic memory retrieval. Cortex. 2018:107:238–250. 28802589 10.1016/j.cortex.2017.07.012 PMC5785567

[ref89] Rugg MD, Vilberg KL. Brain networks underlying episodic memory retrieval. Curr Opin Neurobiol. 2013:23:255–260. 23206590 10.1016/j.conb.2012.11.005 PMC3594562

[ref90] Schapiro AC, McClelland JL, Welbourne SR, Rogers TT, Lambon Ralph MA. Why bilateral damage is worse than unilateral damage to the brain. J Cogn Neurosci. 2013:25:2107–2123. 23806177 10.1162/jocn_a_00441

[ref91] Shine JM, Bissett PG, Bell PT, Koyejo O, Balsters JH, Gorgolewski KJ, Moodie CA, Poldrack RA. The dynamics of functional brain networks: integrated network states during cognitive task performance. Neuron. 2016:92:544–554. 27693256 10.1016/j.neuron.2016.09.018 PMC5073034

[ref92] Stalnaker TA, Cooch NK, Schoenbaum G. What the orbitofrontal cortex does not do. Nat Neurosci. 2015:18:620–627. 25919962 10.1038/nn.3982 PMC5541252

[ref93] Stefaniak JD, Halai AD, Lambon Ralph MA. The neural and neurocomputational bases of recovery from post-stroke aphasia. Nat Rev Neurol. 2020:16:43–55. 31772339 10.1038/s41582-019-0282-1

[ref94] Terzian H, Ore GD. Syndrome of Kluver and Bucy; reproduced in man by bilateral removal of the temporal lobes. Neurology. 1955:5:373–380. 14383941 10.1212/wnl.5.6.373

[ref95] Thompson-Schill SL, D'Esposito M, Aguirre GK, Farah MJ. Role of left inferior prefrontal cortex in retrieval of semantic knowledge: a reevaluation. Proc Natl Acad Sci U S A. 1997:94:14792–14797. 9405692 10.1073/pnas.94.26.14792 PMC25116

[ref96] Uddin LQ . Salience network of the human brain. London, United Kingdom: Academic Press is an imprint of Elsevier. 2017:1–34.

[ref97] Uddin LQ, Supekar K, Amin H, Rykhlevskaia E, Nguyen DA, Greicius MD, Menon V. Dissociable connectivity within human angular gyrus and intraparietal sulcus: evidence from functional and structural connectivity. Cereb Cortex. 2010:20:2636–2646. 20154013 10.1093/cercor/bhq011 PMC2951845

[ref98] Ueno T, Saito S, Rogers TT, Lambon Ralph MA. Lichtheim 2: synthesizing aphasia and the neural basis of language in a neurocomputational model of the dual dorsal-ventral language pathways. Neuron. 2011:72:385–396. 22017995 10.1016/j.neuron.2011.09.013

[ref99] Ullman MT . Is Broca's area part of a basal ganglia thalamocortical circuit? Cortex. 2006:42:480–485. 16881254 10.1016/s0010-9452(08)70382-4

[ref38] van den Heuvel MP, Sporns O. Network hubs in the human brain. Trends Cogn Sci. 2013:17:683–696. 24231140 10.1016/j.tics.2013.09.012

[ref100] Vossel S, Geng JJ, Fink GR. Dorsal and ventral attention systems: distinct neural circuits but collaborative roles. Neuroscientist. 2014:20:150–159. 23835449 10.1177/1073858413494269 PMC4107817

[ref101] Wagner AD, Shannon BJ, Kahn I, Buckner RL. Parietal lobe contributions to episodic memory retrieval. Trends Cogn Sci. 2005:9:445–453. 16054861 10.1016/j.tics.2005.07.001

[ref102] Wagner IC, van Buuren M, Kroes MC, Gutteling TP, van der Linden M, Morris RG, Fernandez G. Schematic memory components converge within angular gyrus during retrieval. elife. 2015:4:e09668. 26575291 10.7554/eLife.09668 PMC4709269

[ref103] Wahl M, Marzinzik F, Friederici AD, Hahne A, Kupsch A, Schneider GH, Saddy D, Curio G, Klostermann F. The human thalamus processes syntactic and semantic language violations. Neuron. 2008:59:695–707. 18786354 10.1016/j.neuron.2008.07.011

[ref104] Wang J, Conder JA, Blitzer DN, Shinkareva SV. Neural representation of abstract and concrete concepts: a meta-analysis of neuroimaging studies. Hum Brain Mapp. 2010:31:1459–1468. 20108224 10.1002/hbm.20950 PMC6870700

[ref105] Warren JE, Crinion JT, Lambon Ralph MA, Wise RJ. Anterior temporal lobe connectivity correlates with functional outcome after aphasic stroke. Brain. 2009:132:3428–3442. 19903736 10.1093/brain/awp270 PMC2792371

[ref106] Whitney C, Kirk M, O'Sullivan J, Lambon Ralph MA, Jefferies E. The neural organization of semantic control: TMS evidence for a distributed network in left inferior frontal and posterior middle temporal gyrus. Cereb Cortex. 2011:21:1066–1075. 20851853 10.1093/cercor/bhq180 PMC3077429

[ref107] Wirth M, Jann K, Dierks T, Federspiel A, Wiest R, Horn H. Semantic memory involvement in the default mode network: a functional neuroimaging study using independent component analysis. NeuroImage. 2011:54:3057–3066. 20965253 10.1016/j.neuroimage.2010.10.039

[ref108] Zanto TP, Gazzaley A. Fronto-parietal network: flexible hub of cognitive control. Trends Cogn Sci. 2013:17:602–603. 24129332 10.1016/j.tics.2013.10.001 PMC3873155

