# Supplementary material for: Distinct but cooperating brain networks supporting semantic cognition
Source: Cereb Cortex. 2022 May 21;33(5):2021–36. doi: 10.1093/cercor/bhac190 (PMC9977382; doi:10.1093/cercor/bhac190)
Supplement: Supplementary_Information_bhac190 [file supplementary_information_bhac190.docx]

**Supplementary Information**

Supplementary Figure 1

Supplementary Figure 2

Supplementary Table 1

Supplementary Table 2

Supplementary Table 3

**Supplementary Figure 1**

**
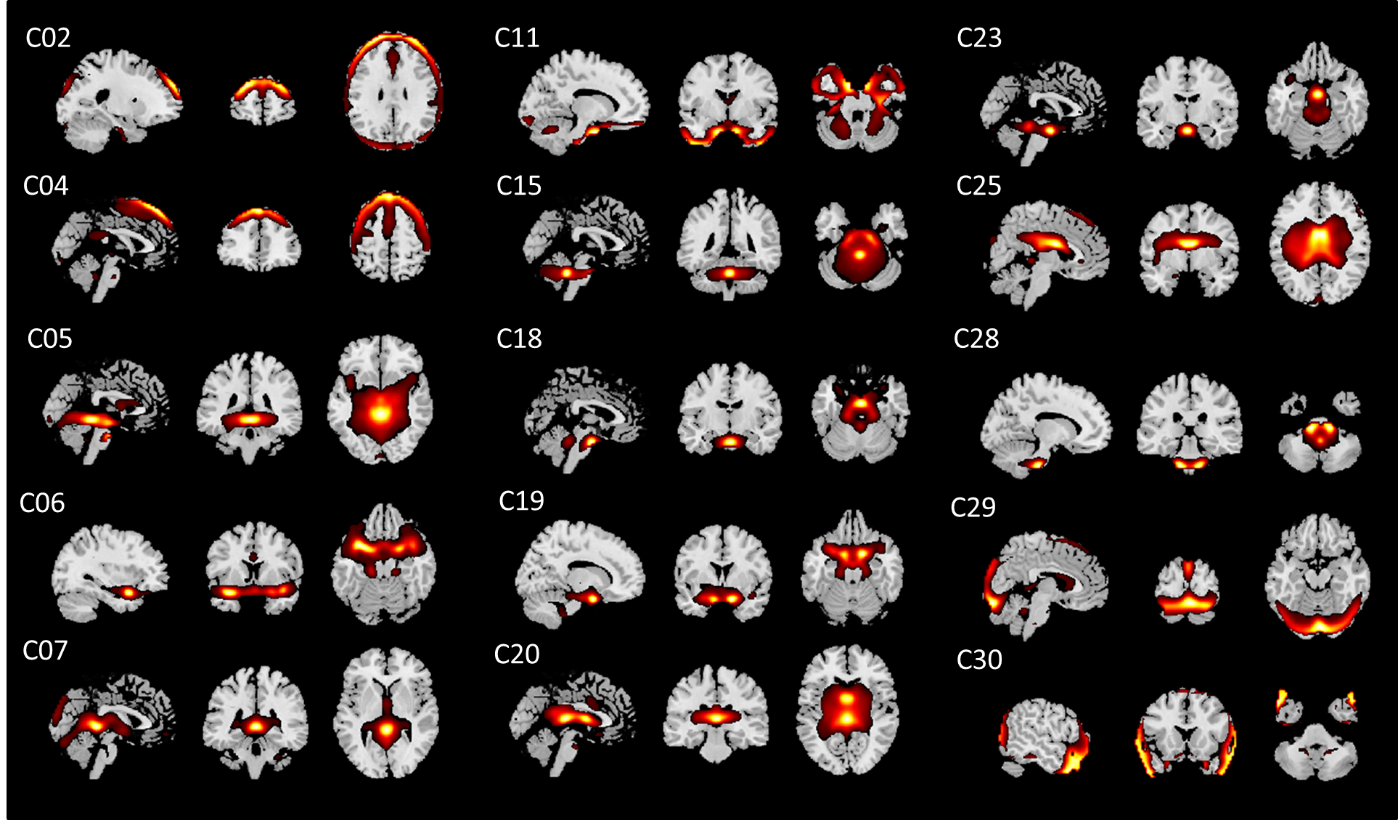
**

**Figure S1**. 15 noise components related to residual artifact including the signal distributed around the edge of the brain and within cerebrospinal fluid spaces, variation in head size, or vascular blood flow.

**Supplementary Figure 2**


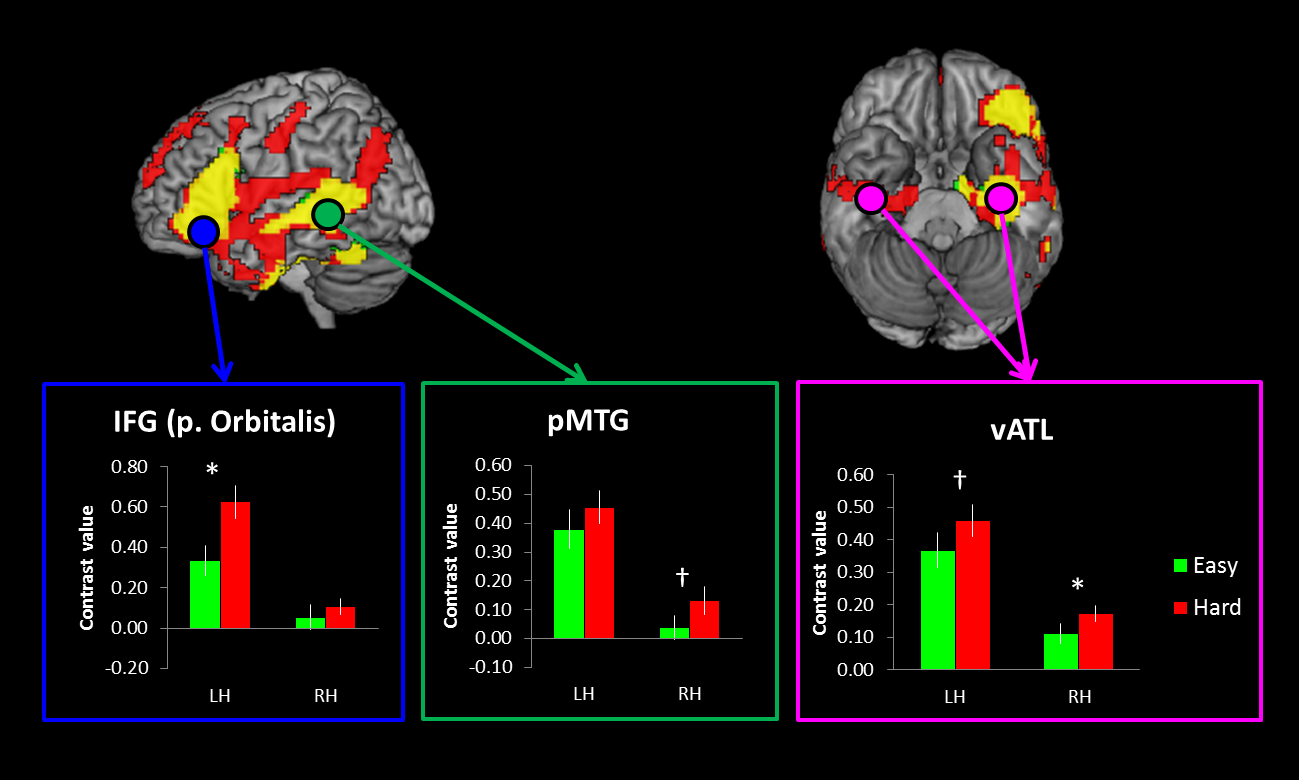


**Figure S2**. The results of ROI analysis in the semantic regions. The green bar indicates the contrast value during the easy semantic condition and the red bar indicates that during the hard semantic condition. * p < 0.05, † p = 0.07.

LH: left hemisphere; RH: right hemisphere

**Supplementary Table 1**

| Components | Brain region | cluster | t | x | y | z |
| --- | --- | --- | --- | --- | --- | --- |
| C01: SN | L vATL | 999 | 15.66 | -33 | -12 | -36 |
|  | L FG |  | 13.22 | -48 | -63 | -21 |
|  | L hippocampus |  | 9.69 | -24 | -18 | -21 |
|  | L IFG (p. Triangularis) | 589 | 17.13 | -48 | 27 | 18 |
|  | L IFG (p. Orbitalis) |  | 8.81 | -33 | 33 | -9 |
|  | L Precentral Gyrus |  | 9.45 | -45 | 3 | 30 |
|  | R Inferior Occipital Gyrus | 452 | 12.47 | 39 | -84 | -12 |
|  | R Inferior Temporal Gyrus |  | 10.74 | 48 | -72 | -12 |
|  | R Fusiform Gyrus |  | 9.75 | 42 | -60 | -21 |
|  | L Precuneus | 284 | 14.74 | -9 | -51 | 12 |
|  | R Middle Frontal Gyrus | 236 | 8.89 | 51 | 27 | 42 |
|  | R IFG (p. Triangularis) |  | 8.63 | 48 | 21 | 24 |
|  | R IFG (p. Opercularis) |  | 7.48 | 48 | 12 | 33 |
|  | SMA | 129 | 12.18 | -3 | 6 | 48 |
|  | L Precentral Gyrus | 104 | 8.92 | -42 | -18 | 60 |
| C03: ESN | L Superior Medial Gyrus | 4184 | 25.36 | -6 | 51 | 36 |
|  | R Superior Medial Gyrus |  | 19.91 | 6 | 57 | 9 |
|  | L Superior Frontal Gyrus |  | 19.29 | -15 | 54 | 33 |
|  | L ACC |  | 15.34 | -6 | 48 | 15 |
|  | R ACC |  | 15.05 | 6 | 51 | 24 |
|  | L lateral ATL | 1028 | 15.11 | -60 | -9 | -21 |
|  | L IFG (p. Orbitalis) |  | 11.6 | -36 | 21 | -15 |
|  | L Inferior Temporal Gyrus |  | 11.03 | -54 | -3 | -33 |
|  | L IFG (p. Triangularis) |  | 6.31 | -48 | 24 | 0 |
|  | R lateral ATL | 507 | 12.67 | 57 | -6 | -24 |
|  | L PCC | 334 | 12.78 | -3 | -51 | 24 |
|  | R Superior Occipital Gyrus | 239 | 10.99 | 21 | -93 | 6 |
|  | R Inferior Occipital Gyrus |  | 7.06 | 36 | -93 | -9 |
|  | L MCC | 237 | 10.38 | 0 | -18 | 36 |
|  | R MCC |  | 5.24 | 9 | -12 | 51 |
|  | L Middle Occipital Gyrus | 205 | 8.6 | -18 | -99 | 6 |
|  | L AG | 154 | 9.58 | -51 | -66 | 30 |
| C08: R. FPN | R IFG (p. Triangularis) | 3822 | 23.62 | 48 | 30 | 24 |
|  | R IFG (p. Opercularis) |  | 19.73 | 51 | 21 | 33 |
|  | R Middle Orbital Gyrus |  | 17.08 | 36 | 57 | -6 |
|  | R Superior Orbital Gyrus |  | 16.09 | 33 | 60 | -3 |
|  | R Middle Frontal Gyrus |  | 15.61 | 36 | 57 | 6 |
|  | L Cerebelum | 554 | 14.6 | -30 | -66 | -33 |
|  | R SupraMarginal Gyrus | 530 | 17.87 | 54 | -45 | 45 |
|  | R Angular Gyrus |  | 11.85 | 48 | -60 | 36 |
|  | R Middle Temporal Gyrus | 259 | 13.52 | 66 | -27 | -9 |
|  | L Middle Orbital Gyrus | 210 | 13.79 | -42 | 57 | -9 |
|  | R MCC | 159 | 10.52 | 6 | -42 | 36 |
| C09: vlPFC-IPC N | R Insula Lobe | 661 | 14.41 | 42 | 3 | -3 |
|  | R Putamen |  | 13.27 | 33 | 0 | -9 |
|  | R IFG (p. Orbitalis) |  | 12.75 | 48 | 24 | -9 |
|  | L Superior Temporal Gyrus | 522 | 16.35 | -45 | -6 | -6 |
|  | L Insula Lobe |  | 15.23 | -33 | 18 | -12 |
|  | L Thalamus | 336 | 9.69 | -3 | -18 | 15 |
|  | L SupraMarginal Gyrus | 166 | 10.33 | -60 | -42 | 36 |
|  | L Inferior Parietal Lobule |  | 9.28 | -54 | -45 | 45 |
| C10: L. FPN | L IFG (p. Orbitalis) | 2723 | 20.92 | -45 | 36 | -3 |
|  | L Middle Frontal Gyrus |  | 20.5 | -42 | 12 | 48 |
|  | L IFG (p. Triangularis) |  | 18.77 | -45 | 27 | 3 |
|  | L Superior Medial Gyrus |  | 15.02 | -9 | 45 | 39 |
|  | L Angular Gyrus | 1045 | 19.97 | -39 | -63 | 36 |
|  | L Inferior Parietal Lobule |  | 15.87 | -54 | -54 | 39 |
|  | R Cerebelum | 547 | 20.12 | 27 | -69 | -30 |
|  | L Middle Temporal Gyrus | 530 | 14.48 | -63 | -39 | -6 |
| C12: R. Rolandic Operculum | R Caudate Nucleus | 3550 | 19.55 | 21 | -18 | 24 |
|  | R Insula Lobe |  | 19.29 | 36 | -12 | 15 |
|  | R Rolandic Operculum |  | 18.03 | 45 | -12 | 15 |
|  | R Postcentral Gyrus |  | 15.03 | 45 | -15 | 36 |
|  | R Precentral Gyrus |  | 12.17 | 51 | -9 | 48 |
| C13: VN1 | R Calcarine Gyrus | 3531 | 22.23 | 15 | -63 | 12 |
|  | R Linual Gyrus |  | 21.64 | 3 | -72 | 3 |
|  | L Linual Gyrus |  | 20.69 | -3 | -75 | 3 |
|  | L Calcarine Gyrus |  | 19.56 | -15 | -63 | 9 |
| C14: Cerebellum | L Cerebelum | 3312 | 23.96 | -24 | -51 | -24 |
|  | R Cerebelum |  | 17.23 | 12 | -75 | -21 |
| C16: MN | R SMA | 5787 | 19.47 | 9 | -18 | 51 |
|  | L SMA |  | 16.6 | 0 | 0 | 48 |
|  | L Precentral Gyrus |  | 16.72 | -27 | -24 | 63 |
|  | R Precentral Gyrus |  | 12.23 | 53 | -19 | 45 |
| C17: mPFC | R ACC | 3640 | 36.95 | 6 | 39 | 3 |
|  | L Superior Orbital Gyrus |  | 30.48 | -15 | 36 | -21 |
|  | L Mid Orbital Gyrus |  | 23.82 | -12 | 39 | -12 |
|  | R Rectal Gyrus |  | 23.2 | 3 | 42 | -15 |
|  | L Rectal Gyrus |  | 22.69 | -9 | 21 | -21 |
|  | R Mid Orbital Gyrus |  | 19.21 | 3 | 27 | -12 |
| C21: VN2 | R Fusiform Gyrus | 4222 | 27.18 | 24 | -84 | -12 |
|  | L Inferior Occipital Gyrus |  | 25.37 | -27 | -78 | -9 |
|  | L Middle Occipital Gyrus |  | 21.69 | -21 | -93 | 6 |
|  | R Middle Occipital Gyrus |  | 21.02 | 33 | -81 | 15 |
| C22: PSN | L Rolandic Operculum | 2246 | 23.36 | -42 | -27 | 18 |
|  | L Superior Temporal Gyrus |  | 19.22 | -42 | -36 | 21 |
|  | L Insula Lobe |  | 17.51 | -33 | -18 | 12 |
|  | R Superior Temporal Gyrus | 1651 | 19.04 | 60 | -9 | 6 |
|  | R SupraMarginal Gyrus |  | 18.79 | 48 | -30 | 24 |
|  | R Rolandic Operculum |  | 18.41 | 63 | -18 | 15 |
|  | R Insula Lobe |  | 17.91 | 42 | -15 | 15 |
| C24: DMN | L Precuneus | 5152 | 33.09 | -3 | -66 | 27 |
|  | R Precuneus |  | 27.57 | 9 | -63 | 27 |
|  | L Angular Gyrus |  | 24.42 | -48 | -63 | 24 |
|  | R Angular Gyrus |  | 19.44 | 45 | -63 | 24 |
|  | R Superior Medial Gyrus | 976 | 19.06 | 6 | 48 | 6 |
|  | L Superior Medial Gyrus |  | 11.76 | 3 | 48 | 21 |
| C26: Salience N | L ACC | 2616 | 20.46 | -6 | 39 | 18 |
|  | R ACC |  | 17.08 | 6 | 42 | 18 |
|  | L Middle Frontal Gyrus |  | 14.63 | -27 | 42 | 24 |
|  | L SMA |  | 12.23 | 3 | 12 | 48 |
|  | L Insula Lobe | 959 | 24.97 | -42 | 9 | -6 |
|  | R Insula Lobe | 915 | 21.63 | 39 | 18 | -3 |
|  | R Caudate Nucleus | 482 | 10.38 | 15 | 0 | 15 |
|  | L Caudate Nucleus |  | 9.12 | -12 | 9 | 6 |
| C27: Thalamus | L Thalamus | 2991 | 17.5 | -9 | -9 | 0 |
|  | L Pallidum |  | 15.99 | -21 | 0 | -3 |

Table S1 The results of ICA

**Supplementary Table 2**

|  | Semantic Easy | | Semantic Hard | | Control Easy | | Control Hard | |
| --- | --- | --- | --- | --- | --- | --- | --- | --- |
| IC | T-value | p | T-value | p | T-value | p | T-value | p |
| C01 | **18.76** | **0.0000** | **6.26** | **0.0000** | **-9.66** | **0.0000** | **13.69** | **0.0000** |
| C03 | **-16.79** | **0.0000** | **21.61** | **0.0000** | **8.45** | **0.0000** | **-17.06** | **0.0000** |
| C08 | **-6.34** | **0.0000** | **6.89** | **0.0000** | **-10.46** | **0.0000** | **12.49** | **0.0000** |
| C09 | **-4.75** | **0.0000** | **10.66** | **0.0000** | -1.00 | 0.3284 | 1.12 | 0.2729 |
| C10 | **11.36** | **0.0000** | **21.05** | **0.0000** | -1.02 | 0.3206 | -0.98 | 0.3394 |
| C12 | **-4.08** | **0.0000** | **21.72** | **0.0000** | **-14.01** | **0.0000** | **14.51** | **0.0000** |
| C13 | **-3.61** | **0.0000** | **7.68** | **0.0000** | **12.27** | **0.0000** | **9.73** | **0.0000** |
| C14 | 1.63 | 0.1181 | **5.16** | **0.0000** | **-11.81** | **0.0000** | **19.97** | **0.0000** |
| C16 | **-16.15** | **0.0000** | **19.83** | **0.0000** | **-5.68** | **0.0000** | **9.70** | **0.0000** |
| C17 | -2.01 | 0.0574 | 1.78 | 0.0891 | **-3.96** | **0.0007** | 0.12 | 0.9024 |
| C21 | **-6.73** | **0.0000** | **4.99** | **0.0000** | **16.40** | **0.0000** | **13.08** | **0.0000** |
| C22 | **-14.85** | **0.0000** | **28.08** | **0.0000** | 1.25 | 0.2251 | -1.27 | 0.2161 |
| C24 | -1.72 | 0.0998 | **-6.59** | **0.0000** | **30.69** | **0.0000** | **-26.06** | **0.0000** |
| C26 | **4.22** | **0.0000** | **17.12** | **0.0000** | **-6.24** | **0.0000** | **6.27** | **0.0000** |
| C27 | **-3.70** | **0.0012** | **4.03** | **0.0006** | -0.84 | 0.4118 | 2.10 | 0.0472 |

Table S2 The ICA results of spatial regression. Bold indicates the significant results from one-sample t-tests. P _FDR-corrected_ < 0.005

**Supplementary Table 3**

| Contrast | Brain region | cluster | t | x | y | z |
| --- | --- | --- | --- | --- | --- | --- |
| Easy | L IFG (p. Triangularis) | 1128 | 9.48 | -48 | 24 | 18 |
| Semantic > control | L IFG (p. Orbitalis) |  | 8.22 | -39 | 39 | -12 |
|  | L IFG (p. Orbitalis) |  | 6.88 | -45 | 36 | -3 |
|  | L IFG (p. Opercularis) |  | 6.41 | -39 | 12 | 30 |
|  | L pMTG | 461 | 5.69 | -66 | -42 | 6 |
|  | L pMTG |  | 5.46 | -60 | -54 | 6 |
|  | L aMTG |  | 4.25 | -57 | -9 | -9 |
|  | L aMTG |  | 4.16 | -60 | -15 | -6 |
|  | L vATL | 441 | 7.09 | -33 | -12 | -36 |
|  | L FG |  | 5.75 | -45 | -54 | -21 |
|  | L FG |  | 5.18 | -36 | -42 | -24 |
|  | R Rolandic Operculum | 205 | 5.57 | 42 | -24 | 21 |
|  | R IPL |  | 5.49 | 45 | -33 | 21 |
|  | L Hippocampus | 103 | 6.25 | -15 | -9 | -15 |
| Hard | L IFG (p. Orbitalis) | 3689 | 11.43 | -36 | 36 | -12 |
| Semantic > control | L IFG (p. Triangularis) |  | 11.33 | -51 | 24 | 18 |
|  | L IFG (p. Orbitalis) |  | 9.46 | -48 | 36 | -3 |
|  | L vATL |  | 7.78 | -33 | -12 | -39 |
|  | L Hippocampus |  | 7.13 | -21 | -12 | -15 |
|  | L pMTG |  | 6.87 | -60 | -57 | 6 |
|  | L pMTG |  | 6.72 | -66 | -45 | 6 |
|  | L AG |  | 6.6 | -54 | -69 | 30 |
|  | L pSTG |  | 6.49 | -51 | -42 | 12 |
|  | R IPL | 1106 | 8.54 | 42 | -33 | 21 |
|  | R Rolandic Operculum |  | 7.84 | 42 | -18 | 18 |
|  | R AG |  | 7.13 | 54 | -66 | 36 |
|  | R ParaHippocampal Gyrus |  | 6.47 | 21 | -15 | -18 |
|  | R Precentral Gyrus |  | 6.35 | 57 | -9 | 42 |
|  | R Middle Temporal Gyrus |  | 5.86 | 60 | -60 | 21 |
|  | R vATL |  | 4.49 | 39 | -15 | -33 |
|  | L Superior Medial Gyrus | 829 | 7.44 | -6 | 39 | 48 |
|  | L Superior Frontal Gyrus |  | 6.76 | -12 | 57 | 27 |
|  | R Superior Medial Gyrus |  | 5.69 | 9 | 45 | 39 |
|  | R MCC | 640 | 5.49 | 9 | -12 | 48 |
|  | L Paracentral Lobule |  | 4.49 | -12 | -18 | 69 |
|  | L MCC |  | 4.25 | -3 | -12 | 45 |
|  | L Postcentral Gyrus | 170 | 5.91 | -51 | -15 | 42 |
|  | L Precentral Gyrus |  | 4.27 | -30 | -30 | 63 |
| Easy | R Middle Occipital Gyrus | 7662 | 13.3 | 30 | -84 | 15 |
| Control > semantic | L Middle Occipital Gyrus |  | 12.4 | -21 | -96 | 9 |
|  | R Fusiform Gyrus |  | 12.37 | 30 | -66 | -12 |
|  | R Calcarine Gyrus |  | 11.96 | 15 | -96 | 3 |
|  | R Precuneus |  | 10.55 | 18 | -54 | 18 |
|  | L Fusiform Gyrus |  | 10.55 | -24 | -72 | -12 |
|  | R Linual Gyrus |  | 10.05 | 12 | -87 | -12 |
|  | L Superior Occipital Gyrus |  | 8.53 | -21 | -75 | 30 |
|  | R Superior Frontal Gyrus | 166 | 3.63 | 27 | 11 | 65 |
|  | L Middle Frontal Gyrus | 108 | 5.26 | -24 | 0 | 54 |
| Hard | R Middle Occipital Gyrus | 8893 | 16.13 | 36 | -78 | 15 |
| Control > semantic | R Fusiform Gyrus |  | 14.61 | 30 | -66 | -12 |
|  | L Middle Occipital Gyrus |  | 12.68 | -21 | -81 | 21 |
|  | R Precuneus |  | 11.95 | 18 | -54 | 18 |
|  | L Fusiform Gyrus |  | 11.89 | -24 | -72 | -12 |
|  | R IFG (p. Opercularis) | 727 | 7.5 | 51 | 6 | 27 |
|  | R Superior Frontal Gyrus |  | 7.4 | 33 | 3 | 63 |
|  | R Precentral Gyrus |  | 3.97 | 45 | 3 | 45 |
|  | L Middle Frontal Gyrus | 332 | 9.04 | -24 | -3 | 51 |
|  | R Superior Orbital Gyrus | 178 | 5.48 | 18 | 18 | -21 |
|  | R Insula Lobe |  | 4.06 | 30 | 21 | -15 |
|  | R Middle Orbital Gyrus |  | 3.78 | 27 | 45 | -18 |
| Interaction | R Middle Occipital Gyrus | 715 | 31.82 | 30 | -72 | 30 |
|  | R IPS |  | 27.39 | 30 | -48 | 39 |
|  | R Inferior Temporal Gyrus |  | 26.71 | 48 | -63 | -12 |
|  | R Middle Temporal Gyrus |  | 24.66 | 45 | -72 | 15 |
|  | R Precuneus |  | 18.72 | 18 | -66 | 42 |
|  | R Fusiform Gyrus |  | 17.06 | 36 | -51 | -12 |
|  | L Superior Medial Gyrus | 557 | 35.74 | -6 | 42 | 48 |
|  | L Mid Orbital Gyrus |  | 23.1 | 3 | 63 | -12 |
|  | L vmPFC |  | 22.94 | -18 | 36 | 42 |
|  | R vmPFC |  | 22.68 | 6 | 63 | -6 |
|  | R Superior Medial Gyrus |  | 21.79 | 9 | 45 | 39 |
|  | L Superior Medial Gyrus |  | 18.99 | 0 | 63 | 6 |
|  | L Precuneus | 443 | 33.89 | -9 | -51 | 9 |
|  | R Precuneus |  | 29.44 | 6 | -51 | 27 |
|  | L PCC |  | 19.99 | -3 | -42 | 33 |
|  | L AG | 206 | 206 | -48 | -66 | 30 |
|  | L IFG (p. Orbitalis) | 202 | 26.13 | -33 | 33 | -15 |
|  | L IFG (p. Triangularis) |  | 15.66 | -45 | 33 | 0 |
|  | R Middle Frontal Gyrus | 100 | 23.64 | 36 | 3 | 63 |

Table S3 The results of GLM analysis
